# Gene expression variation underlying tissue-specific responses to copper stress in *Drosophila melanogaster*

**DOI:** 10.1093/g3journal/jkae015

**Published:** 2024-01-23

**Authors:** Elizabeth R Everman, Stuart J Macdonald

**Affiliations:** School of Biological Sciences, The University of Oklahoma, 730 Van Vleet Oval, Norman, OK 73019, USA; Molecular Biosciences, University of Kansas, 1200 Sunnyside Ave, Lawrence, KS 66045, USA

**Keywords:** heavy metals, gene expression, eQTL mapping, DSPR

## Abstract

Copper is one of a handful of biologically necessary heavy metals that is also a common environmental pollutant. Under normal conditions, copper ions are required for many key physiological processes. However, in excess, copper results in cell and tissue damage ranging in severity from temporary injury to permanent neurological damage. Because of its biological relevance, and because many conserved copper-responsive genes respond to nonessential heavy metal pollutants, copper resistance in *Drosophila melanogaster* is a useful model system with which to investigate the genetic control of the heavy metal stress response. Because heavy metal toxicity has the potential to differently impact specific tissues, we genetically characterized the control of the gene expression response to copper stress in a tissue-specific manner in this study. We assessed the copper stress response in head and gut tissue of 96 inbred strains from the *Drosophila* Synthetic Population Resource using a combination of differential expression analysis and expression quantitative trait locus mapping. Differential expression analysis revealed clear patterns of tissue-specific expression. Tissue and treatment specific responses to copper stress were also detected using expression quantitative trait locus mapping. Expression quantitative trait locus associated with *MtnA*, *Mdr49*, *Mdr50*, and *Sod3* exhibited both genotype-by-tissue and genotype-by-treatment effects on gene expression under copper stress, illuminating tissue- and treatment-specific patterns of gene expression control. Together, our data build a nuanced description of the roles and interactions between allelic and expression variation in copper-responsive genes, provide valuable insight into the genomic architecture of susceptibility to metal toxicity, and highlight candidate genes for future functional characterization.

## Introduction

Many forms of stress resistance contribute to the overall health of the individual, both through immediate consequences brought on by direct exposure to the stressor, and indirectly by increasing the risk of experiencing deleterious consequences in the future. Heavy metals are one such type of stressor. Broadly, exposure to heavy metals results in oxidative stress due to the reactive state of metal ions (Stohs and Bagchi 1995; Ercal *et al.* 2001; Jomova *et al.* 2010). Acute exposure can cause gastrointestinal distress and vomiting as well as damage to intestinal lining (Taylor *et al.* 2020; Gerhardsson 2022). Chronic heavy metal exposure has been linked to neurodegenerative diseases in humans including Alzheimer's and Parkinson's Diseases in adults (Bonilla-Ramirez *et al.* 2011; Chen, Miah *et al*. 2016; Bagheri *et al.* 2018; Liddell and White 2018; Bisaglia and Bubacco 2020) and learning and behavioral disorders in developing individuals (Jomova *et al.* 2010; Blaurock-Busch *et al.* 2011; Hsueh *et al.* 2017; Lee *et al.* 2018). Additionally, exposure can increase morbidity associated with health conditions including multiple sclerosis and osteoporosis (Åkesson *et al.* 2006) as well as anemia (Turgut *et al.* 2007). Heavy metal exposure risk is often related to occupation (Castilla and Nealler 1978; Neuberger *et al.* 1990; World Health Organization 1996; Khan *et al.* 2008; Karnchanawong and Limpiteeprakan 2009; He *et al.* 2015; Knoblauch *et al.* 2017), and like many diseases [e.g. type 2 diabetes, Crohn's disease, heart disease, IBD (http://www.genome.gov/gwasstudies) (Hindorff *et al.* 2009; Plomin *et al.* 2009)], susceptibility to metal stress is a genetically complex trait (Zhou *et al.* 2016; Everman *et al.* 2021, 2023).

Quantitative trait locus (QTL) mapping has provided a powerful means of characterizing allelic variation that contributes to lead, cadmium, copper, and zinc resistance in several model systems including worms, flies, yeast, and plants (MacNair 1983; Jones *et al.* 2006; Bálint *et al.* 2007; Courbot *et al.* 2007; Ruden *et al.* 2009; Ehrenreich *et al.* 2012; Evans *et al.* 2018; Everman *et al.* 2021). The *Drosophila melanogaster* model system has been leveraged in several of these studies to examine the genetic architecture of metal stress response. It is an excellent model because all of the major genes that are involved in metal detoxification play similar roles in humans, allowing for the insight gained from work in *Drosophila* to have broader applications for understanding the impact of heavy metals on human health (Balamurugan *et al.* 2004; Egli, Domènech *et al*. 2006; Egli, Yepsikoposyan *et al*. 2006; Turski and Thiele 2007; Burke *et al.* 2008; Hatori and Lutsenko 2013; Calap-Quintana *et al.* 2017; Zhou *et al.* 2017). Genome-wide association mapping in flies revealed multiple SNPs that are associated with resistance to lead and cadmium toxicity (Zhou *et al.* 2016, 2017), and we previously demonstrated that resistance to copper toxicity is influenced by multiple QTL that included several conserved genes involved in copper metabolism and homeostasis (Everman *et al.* 2021). Collectively, these studies have provided valuable insight into naturally occurring genetic variants that contribute to variation in resistance to several common heavy metal pollutants. However, most studies that examine the genetic control of heavy metal resistance have focused either on the whole-animal response or on single tissues. Furthermore, work over the last decade has clearly demonstrated that the majority of sites that influence trait variation are more likely to have impacts on gene regulation rather than to result in protein coding changes (Pickrell 2014; Zhang and Lupski 2015; reviewed in Boyle *et al.* 2017; Alsheikh *et al.* 2022). Therefore, an important opportunity remains to characterize the genetic control of the gene expression response to heavy metal stress and to determine the degree to which this genetic control is tissue specific.

Transcriptomics studies have repeatedly demonstrated that exposure to heavy metal stressors increases expression of several gene families involved in metal detoxification, metabolism, and transport as well as oxidative stress response (González *et al.* 2008; Catalán *et al.* 2016; Calap-Quintana *et al.* 2017; Qu *et al.* 2018; Everman *et al.* 2021; Felmlee *et al.* 2022; Green *et al.* 2022). However, the gene expression response to metal stress is itself also subject to genetic variation in the regulation of the transcriptional response, i.e. different genotypes can vary in their response to the same exposure (Findley *et al.* 2021). The loci that contribute to variation in the gene expression response can be identified using expression QTL (eQTL) mapping (e.g. Jansen 2001; De Koning and Haley 2005; Rockman and Kruglyak 2006; Gilad *et al.* 2008). We combined RNA sequencing and eQTL mapping to examine the tissue-specific genetic control of the gene expression response to the common metal pollutant copper using the *D. melanogaster* model system.

Although a broad oxidative stress response is expected across tissues in response to metal stress, copper is one of several heavy metals that have the potential to elicit different gene expression responses in neurological and gut tissue. This expected tissue-specific difference is in part due to the spatial distribution of specialized copper accumulation cells found in the gut of most animals including flies and humans (Calap-Quintana *et al.* 2017; Miguel-Aliaga *et al.* 2018). Genes that are responsible for maintaining normal homeostasis of the set of essential heavy metals (e.g. copper and zinc), and that play a role in the expulsion of toxic metals such as cadmium and lead, are primarily expressed in these specialized cells where they play an important protective role against excess metal ions (Calap-Quintana *et al.* 2017). Due to the link between neurological disease and heavy metal exposure in humans (Chen, Miah *et al*. 2016), we assay both head and gut tissue using copper as a model metal.

Our study leverages the *Drosophila* Synthetic Population Resource (DSPR, King, Macdonald *et al*. 2012), a panel of multiparental, advanced intercross strains that has been used in many mapping studies to characterize the complex genetic architecture underlying quantitative traits (e.g. Kislukhin *et al.* 2013; Najarro *et al.* 2015; Highfill *et al.* 2017; Everman *et al.* 2019; Williams-Simon *et al.* 2019; Shahrestani *et al.* 2021). We recently demonstrated that the DSPR strains exhibit dramatic variation in copper resistance, and we identified numerous QTL associated with copper resistance (Everman *et al.* 2021). Here, we build on this study using 96 strains chosen based on their estimated copper resistance. Half (48) of the strains are highly resistant, while the other half (48) of the strains are highly sensitive to copper. This design maximizes the phenotypic variation in copper resistance among strains, allowing us to explore the gene expression and regulation patterns in response to copper stress in phenotypically distinct strains. Since the DSPR is composed of genetically stable inbred strains, it is especially well suited to eQTL mapping studies that quantify expression across tissues and treatments.

## Methods

### Fly stocks

We used the DSPR, a large multiparental mapping panel with >1,500 recombinant inbred strains (see King, Macdonald *et al*. 2012, King, Merkes *et al*. 2012 for additional details). In a previous study, we exposed 60 adult females from each of 1,556 strains (767 and 789 strains from the A and B populations, respectively) to 50 mM CuSO_4_, measuring resistance as the percentage of adults alive at 48 h (Supplementary Table 2 in Everman *et al.* 2021). For the present study, we selected 48 of the “resistant” strains from the B population that were in the top 25% of the distribution of adult copper resistance (75.11–100% survival) and 48 of the “sensitive” B population strains from the bottom 25% of the distribution (0–25% survival; Supplementary Fig. 1).

### Rearing and assay conditions

Flies were reared and assayed at 25 °C and 50% humidity with a 12:12 h light:dark photoperiod. To maintain consistency with prior work, experimental females for the present study were obtained from each of the 96 DSPR strains in the same manner used to measure adult copper resistance (Everman *et al.* 2021). Briefly, adults were placed on cornmeal–molasses–yeast media and were allowed to oviposit for 2 days before being discarded. Experimental females from the following generation were sorted over CO_2_ into groups of 20 and allowed to recover for 24 h on fresh cornmeal–molasses–yeast media. Following recovery, 3–5-days-old females were transferred to Instant *Drosophila* Media (Carolina Biological Supply Company 173200) hydrated with either water (control) or 50 mM CuSO_4_ (Copper(II) sulfate, Sigma–Aldrich C1297) and exposed to treatment conditions for 8 h, after which tissues were harvested (see below). The 8-hour exposure period lasted from lights on (8 AM) to 4 h before lights off. No flies died during the 8-hour exposure period.

Strains were assayed in a series of small batches to accommodate the time required for tissue processing following the exposure period, with sensitive and resistant strains evenly distributed across each batch. Given the different processing techniques for heads and gut, we obtained these tissues from different pools of individuals of the same strains in different batches. The average batch size for head collection was 25 DSPR strains and average batch size for gut dissection was 6 DSPR strains.

### Tissue collection, RNA isolation, and sequencing library preparation

To obtain head tissue, we exposed 60 females per DSPR strain to control and copper conditions (three vials of 20 females per treatment) as described above. At the end of the exposure period, flies from each treatment and strain were pooled in a labeled screw-top 50 mL tube (1 tube per strain and treatment), flash frozen in liquid nitrogen, immediately vortexed to separate heads from bodies, and then stored at −80 °C for up to 5 days. We used a series of 3-inch diameter stacking brass sieves—chilled on dry ice for at least 5 min to prevent tissue thawing—to isolate the heads from each sample (see Supplementary Methods (Ericsson 1999)). Heads for each strain/treatment were dispensed into a screw-top microcentrifuge tube containing three to four glass beads and held on dry ice. Subsequently, we added 300 μL TRIzol Reagent (Invitrogen, 15596018) to each sample and stored samples at −80 °C until RNA extraction.

Guts, including the fore-, mid-, and hindgut, were dissected from 10 live individuals per strain/treatment combination in 1× PBS and placed in screw-top microcentrifuge tubes containing three to four glass beads and 300 μL TRIzol. Tissue from the 10 dissected females was pooled into a single sample per strain and treatment. Samples were chilled on ice during dissection and stored at −80 °C until RNA extraction.

RNA was extracted from each of the 384 samples (96 strains × 2 tissues × 2 treatments) in batches of 30 samples using the Direct-zol RNA Miniprep kit (Zymo Research, R2050). Batches consisted of samples from the same tissue and were processed in the order individual samples were collected. RNA was eluted in 50 μL water, and concentrations were determined via a NanoDrop spectrophotometer and then standardized to 20 ng/μL in 96-well plates. Because head and gut tissues were not collected contemporaneously, two plates contained samples from heads and two contained samples from gut tissue. The order of the samples from each strain and treatment was largely consistent across the head and gut sets of plates; all plates included both copper and control samples for a given strain and held an even representation of sensitive and resistant strains (Supplementary Table 1).

Half-reaction, unique dual indexed TruSeq Stranded mRNA sequencing libraries (Illumina, 20020595) were generated and sequenced by the University of Kansas Genome Sequencing Core. Concentrations for every library were obtained using the Qubit dsDNA HS assay kit (ThermoFisher, Q32854), and successful libraries were pooled based on concentration within each of the four tissue-specific plates described above (Supplementary Table 1). This resulted in two pools of head libraries (one 94- and one 96-plex), and two pools of gut libraries (one 93- and one 96-plex). Paired-end 37 bp reads were obtained by sequencing each pool separately on NextSeq550 High-Output flowcells. Raw read counts for the 96-plex Head, 94-plex Head, 93-plex Gut, and 96-plex Gut libraries were 3.8–5.6 million PE37 read pairs per sample (Supplementary Fig. 2). We note that to achieve sufficient reads, the 96-plex Head library pool was sequenced over two flow cells, and the reads were combined for downstream analysis (Supplementary Figs. 2 and 3).

### Sequencing data preprocessing

#### Read alignment and gene filtering

Quality assessment, adapter removal, and read trimming (retaining reads with a minimum of 15 bp) were performed with fastp (v. 0.20.1) (Chen *et al.* 2018). Only paired reads were retained. Filtered read pairs were aligned in a variant-aware manner to the *Drosophila* reference genome (Release 6.33) using HISAT2 (v. 2.1.0) (Kim *et al.* 2019). SNP variants identified in the DSPR founders (http://wfitch.bio.uci.edu/∼tdlong/SantaCruzTracks/DSPR_R6/dm6/variation/DSPR.r6.SNPs.vcf.gz) were included in the HISAT2 genome index following instructions provided with the software. Aligned reads were sorted with SAMtools (v. 1.9) (Li *et al.* 2009) and quantified with featureCounts (v. 2.0.1) (Liao *et al.* 2014). Post-alignment and quantification analyses were all performed in R (v. 4.1.3) (R Core Team 2023). Average library size prior to additional filtering was 4.03 million reads. In R, genes with low expression were filtered out by first removing genes with zero counts across all samples and then by removing genes with fewer than 10 counts in 47 samples (the number of samples unique to each of the three-way interaction categories, e.g. low copper resistance head tissue samples exposed to copper stress; Chen, Lun *et al*. 2016). Following filtering, 9,842 genes were retained for downstream analyses. We note that normalized gene expression counts obtained via kallisto pseudoalignment (v. 0.46.2, Bray *et al.* 2016) generated extremely similar results (see Supplementary Methods).

### Effect of tissue, treatment, and resistance class on gene expression

Gene expression (read counts) were normalized using the weighted trimmed mean of *M*-values (mean of log expression ratios; Robinson and Oshlack 2010), and DE analysis was performed with limma (Ritchie *et al.* 2015) by fitting the full-factorial gene-wise linear model (Normalized Read Counts ∼ Tissue * Treatment * Resistance class + Sample Pooling) (Quinn and Keough 2002; Smyth 2004; Ritchie *et al.* 2015). We employ a “Sample Pooling” term in the model to account for technical variation that is due to plate, library pool, and/or sequencing flowcell, technical factors that are not individually separable in our design. However, because the pairs of head and gut plates do not contain identical subsets of strains, some technical variation is not captured by the “Sample Pooling” term. Genes with expression variation significantly influenced by each term in the model (Treatment, Resistance class, Tissue, Tissue by Treatment, Tissue by Resistance class, Treatment by Resistance class, and Tissue by Treatment by Resistance class) were identified by fitting contrasts to the full model in limma with the contrasts.fit function. This step was followed by the eBayes function, which uses an empirical Bayes method (Efron and Morris 1973; Morris 1983) to calculate log fold change in normalized read counts and to determine significance between each group identified by the contrast (contrast order: expression in gut relative to head tissue, copper relative to control treated samples, sensitive relative to resistant strains). Significant DE was determined using adjusted *P* values to account for multiple comparisons (Benjamini-Hochberg multiple test correction, Benjamini and Hochberg 1995; FDR threshold = 5%) in limma.

### Tissue- and treatment-specific eQTL analysis

We performed eQTL mapping separately for six datasets: Head-Control (9,783 genes), Head-Copper (9,842 genes), Head-Response (9,830 genes), Gut-Control (9,842 genes), Gut-Copper (9,842 genes), and Gut-Response (9,841 genes). The “Response” to copper treatment was calculated as gene expression (modeled as gene counts) under copper conditions minus expression under control conditions for all paired DSPR strain samples. Positive Response values indicate genes with higher expression under copper conditions (copper treatment-induced), while negative Response values indicate genes that are repressed by copper treatment. To preserve the direction of gene expression change in both Response datasets during preparation for eQTL mapping, we performed log2 transformation and quantile normalization on the absolute values of the copper-response read counts and reassigned the sign following normalization. Each dataset was analyzed separately because R/qtl2 (implementation described below, Broman *et al.* 2019) does not allow all datasets to be included in the same model and because the number of expression traits varied slightly among datasets.

Preparation for eQTL analysis of each of the six datasets proceeded with the following steps: 1. Read counts were quantile normalized (Amaratunga and Cabrera 2001; Zhao *et al.* 2020) following log2 transformation (Keene 1995), 2. Technical and environmental factors were accounted for using principal components analysis (PCA, Pearson 1901; Hotelling 1933; Jolliffe 2002); prcomp function in R (R Core Team 2023)) by regressing out the effects of PCs that explained more than 2% of the variance in quantile normalized gene expression or that were correlated with known technical factors (Supplementary Table 2) (Leek and Storey 2007; Pickrell *et al.* 2010; Gaffney *et al.* 2012; King *et al.* 2014), and 3. Residuals were quantile normalized and used as input data for eQTL analyses (referred to as residual counts below). See Supplementary Methods for additional details on preparation steps.

eQTL mapping (Rockman and Kruglyak 2006) was performed on quantile normalized residual gene expression for each of the six datasets using R/qtl2 (Broman *et al.* 2019). eQTL mapping treats each gene expression measure as a trait, and tests for associations between strain (genotype) and variation in gene expression at each genome marker (every 10 kb in the DSPR, King, Macdonald *et al*. 2012). For each gene, eQTL mapping regresses the residual counts on additive probabilities indicating the likelihood that a particular region of the genome is inherited from one of the 8 DSPR founders in a series of scans (one scan per expression trait assessed). R/qtl2 uses Haley–Knott regression to perform each genome scan, fitting each model without a covariate (Haley and Knott 1992; Broman *et al.* 2019). Gene-specific genome wide eQTL significance thresholds were assigned by permuting residual count estimates among the DSPR strains 1,000 times (Churchill and Doerge 1994; Broman *et al.* 2019), and eQTL were defined as peaks surviving a 95% LOD threshold. Peaks were identified using the standard interval mapping approach implemented in the package DSPRqtl (Lander and Botstein 1989; Broman *et al.* 2019) as described in King, Merkes *et al*. (2012).

Given the modest sample size for each eQTL analysis (93–96 DSPR strains), for above-threshold eQTL peaks (described above) we defined QTL confidence intervals using a 3-LOD drop, since a 2-LOD drop can give overly narrow intervals when fewer strains are assayed (King, Macdonald *et al*. 2012). *Cis*-eQTL were defined as above-threshold peaks for which the upper or lower boundary of the 3-LOD drop was within 1.5 cm of the target gene, while peaks outside this interval were classified as distant or *trans*-eQTL. We note that broader peak intervals result in more peaks being designated as *cis*-eQTL and fewer peaks as *trans*-eQTL (King *et al.* 2014; King and Long 2017). Peaks were removed if they consisted of a single, above-threshold marker, or if the peak position was outside the lower and upper peak boundaries (such phenomena are more common in experiments with limited sample size and are often found near telomere and centromere regions where the impact of low power is exacerbated). eQTL peaks, estimated founder effects, and percent variance were identified and calculated using custom code derived from the DSPRqtl (King, Merkes *et al*. 2012) and R/qtl2 mapping programs (see Supplementary data).

We examined the six datasets for evidence of eQTL that were shared among datasets. Comparisons were made between tissues within treatment (Head-Control vs Gut-Control, Head-Copper vs Gut-Copper, Head-Response vs Gut-Response) and within tissue between treatments (Heads-Control vs Heads-Copper and Gut-Control vs Gut-Copper). Our ability to identify shared eQTL across multiple datasets is influenced by power to detect eQTL and the effect size of the eQTL (McKenzie *et al.* 2014). There are many approaches and methods to determine whether eQTL overlap (e.g. Ding *et al.* 2010; McKenzie *et al.* 2014; Van Den Berg *et al.* 2019). To account for increased uncertainty due to power and eQTL effect size, eQTL were considered shared if the peak positions were within 1.5 cm of each other and/or if the peak intervals overlapped. We also note that our results should be interpreted with care given the large number of tests completed for each tissue and treatment combination and the likely inflation of false discovery rates (Colquhoun 2014).

### Gene annotations and ontology analyses

We obtained annotation and ontology information for focal sets of genes highlighted by differential expression (DE) analysis and eQTL mapping using the *D. melanogaster* annotation tool available from biomaRt (v. 2.50.3, Durinck *et al.* 2005) via Ensembl (Martin *et al.* 2023) and the orb.DM.eg.db R package (v. 3.14.0, Carlson 2019). Gene Ontology (GO) enrichment analyses (Ashburner *et al.* 2000; Tomczak *et al.* 2018; Gene Ontology Consortium *et al*. 2023) were performed using the R package GOstats (v. 2.60.0, Falcon and Gentleman 2007), which uses hypergeometric tests for overrepresentation of GO terms with a correction for multiple tests (Subramanian *et al.* 2005).

## Results

RNA sequencing data was obtained for 96 DSPR strains that had been previously assayed for copper resistance, 48 of which showed considerable resistance to copper stress and 48 of which were highly susceptible. Following exposure of animals to copper and to a control treatment, we obtained RNA from head and gut tissue, ultimately generating 384 libraries (96 strains × 2 tissues × 2 treatments = 384 sequencing libraries). Using DE analysis we examined the regulatory effects of resistance class, tissue, and treatment, and we used eQTL mapping to characterize the genetic control of the gene expression response to copper.

### Effect of tissue, treatment, and resistance class on gene expression

A principal goal of our study was to characterize the gut- and head-specific gene expression responses to copper exposure in genetically diverse copper-sensitive and copper-resistant DSPR strains. Our DE model tested the three-way interaction between tissue (gut, head), treatment (control, 8-hour exposure to 50 mM CuSO_4_), and DSPR strain resistance class (resistant, sensitive). The primary model parameters that influenced expression were tissue and treatment. Gene expression was strikingly distinct between gut and head samples resulting in DE of 91% of genes (5% FDR). Although we cannot rule out the potential influence of collecting head and gut samples separately on the distinct effect of tissue we observed, it is unlikely that this is the primary factor resulting in the vast differences in gene expression between tissue types because tissues were obtained from individuals of the same strain. In addition, it has been demonstrated that tissue type can be generally categorized and differentiated by gene expression profiles (Hsiao *et al.* 2001). Treatment alone influenced gene expression in 12% of genes, while resistance class alone influenced expression of three genes: *asRNA:CR44107*, *CG18563* (involved in serine-type endopeptidase activity, Thurmond *et al.* 2019), and *CG6023*.

For 70% of genes, expression was influenced by an interaction between tissue and treatment. The effect of resistance class varied subtly between gut and head tissue, influencing 32% of genes, with the interaction driven by a slightly greater effect of resistance class in gut tissue vs head tissue. The treatment and resistance class interaction did not influence the expression of any gene tested, and the three-way interaction between tissue, treatment, and resistance class influenced 0.13% of genes, of which eight had known or predicted functions. None of the genes influenced by the three-way interaction were known copper response genes or had clear connections to metal toxicity response using gene annotation information provided by Ensembl (Martin *et al.* 2023) and FlyBase (Gramates *et al.* 2022). Gene annotation information on all DE genes is available in the Supplementary data.

#### Differential expression of copper-related genes

**Fig. 1.**
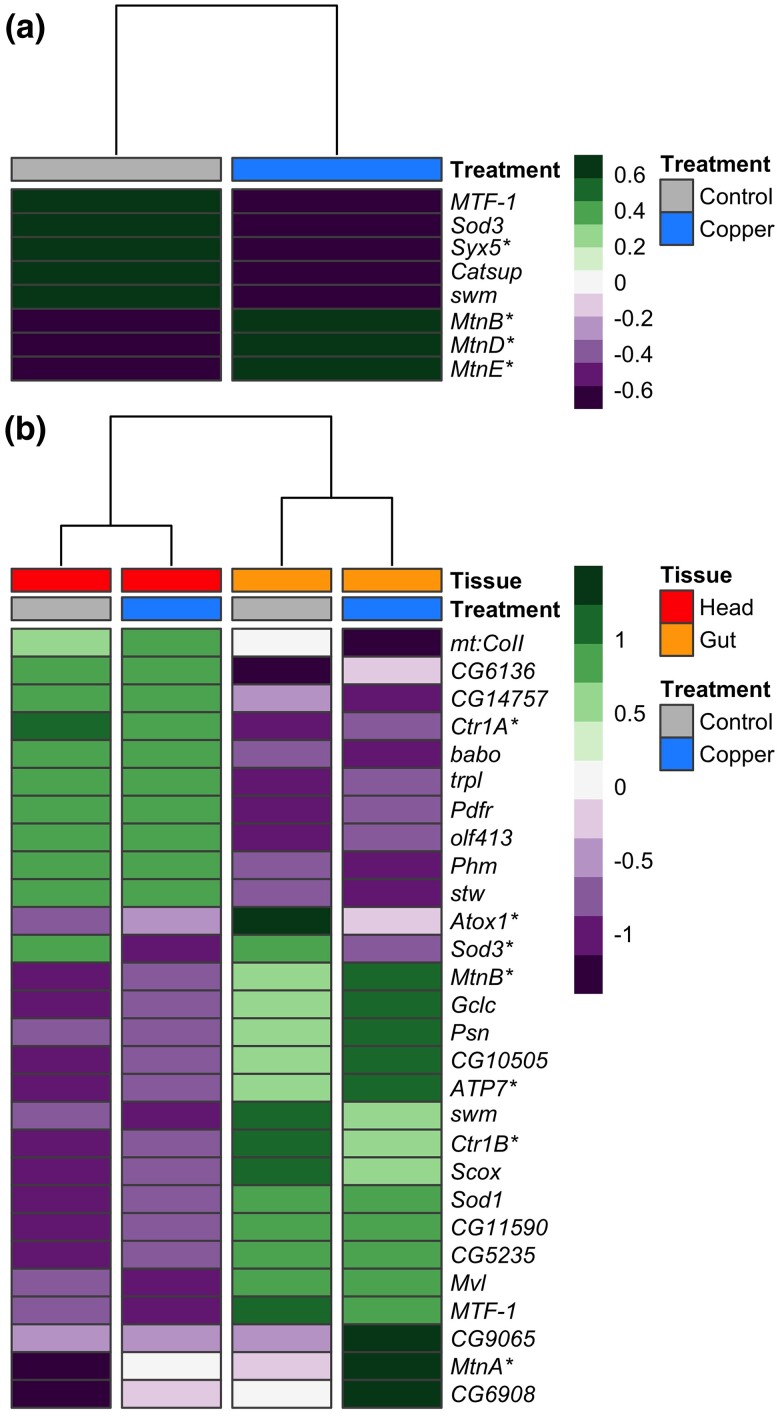
Treatment and the interaction between treatment and tissue influenced expression of several genes previously shown to be involved in detoxification, homeostasis, or binding of copper and other heavy metals. a) Heatmap of genes differentially expressed due to treatment that have been previously associated with copper ion response. b) Heatmap of genes differentially expressed due to the interaction between tissue and treatment. In both heatmaps, tissue and treatment groups are indicated at the top of the heatmaps, and gene expression is presented as average normalized expression for strains belonging to the two resistance classes. Asterisks beside gene names highlight genes discussed in text. Color bar scale indicates *z*-normalized gene expression.

Using gene annotations provided by Ensembl (Martin *et al.* 2023) and FlyBase (Gramates *et al.* 2022), seven genes that have been previously linked to binding, metabolism, and detoxification of copper were among the DE genes influenced by treatment (Fig. 1a) and 28 genes in these categories were influenced by the tissue by treatment interaction (Fig. 1b) (Calap-Quintana *et al.* 2017; Gramates *et al.* 2022; Martin *et al.* 2023). For several genes that have been previously investigated in the context of copper toxicity, the change in expression across treatments/tissues was in the expected direction. For instance, *Syx5* is a critical copper homeostasis gene that is required for proper accumulation of copper ions under normal (copper-scarce) conditions and is hypothesized to aid in proper localization of copper import proteins (Norgate *et al.* 2010). Norgate *et al.* (2010) demonstrated reduced expression of *Syx5* increased resistance to excess copper. Complementing this previous work, we found exposure to copper stress significantly reduced expression of *Syx5* in both head and gut tissues (Fig. 1a), suggesting that downregulation of *Syx5* may result from copper stress in multiple tissue types.

Expression of the metallothionein family genes in response to copper stress was also consistent with expectations. Copper exposure was expected to increase expression of the metallothionein genes, which are involved in the sequestration of excess copper ions as a first defense against copper toxicity (Egli, Yepsikoposyan *et al*. 2006; Calap-Quintana *et al.* 2017). Further, expression was expected to be higher in gut compared to head tissue because primary expression of metallothioneins has been shown to occur in specialized copper accumulating cells that line the midgut in flies (Calap-Quintana *et al.* 2017) As expected, we found that expression of four metallothioneins (*MtnA*, *MtnB*, *MtnD*, and *MtnE*) was significantly increased in response to copper exposure in both head and gut tissues but the increase in expression of *MtnA* and *MtnB* was more pronounced in gut tissue (Fig. 1b).

Three key copper transporters (*Ctr1A*, *Ctr1B*, and *ATP7*) were also among the genes influenced by an interaction between tissue and treatment that followed expected expression patterns based on previous reports. The ATP7 and Ctr1B copper transporters function primarily in specialized copper-accumulating cells that line the intestine in mammals and the midgut in flies (Zhou *et al.* 2003; Turski and Thiele 2007; Calap-Quintana *et al.* 2017), whereas the Ctr1A copper transporter is ubiquitously expressed in both mammals and flies (Turski and Thiele 2007; Calap-Quintana *et al.* 2017). Under control and copper conditions, we observed higher expression of *ATP7* and *Ctr1B* in gut tissue relative to head tissue and higher expression of *Ctr1A* in head tissue compared to gut tissue (Fig. 1b). Our data are also consistent with previous reports that *Ctr1A/B* copper importers are downregulated in *D. melanogaster* in response to copper overexposure (Zhou *et al.* 2003; Calap-Quintana *et al.* 2017) as we observed a decrease in expression of both *Ctr1A* and *Ctr1B* following copper exposure. The pattern of decreased expression in response to copper was consistent across tissue but was more pronounced in head tissue (*Ctr1A*) or gut tissue (*Ctr1B*) depending on the gene. Although *ATP7* expression under control conditions followed tissue-specific expectations, we observed that exposure to copper stress significantly increased *ATP7* expression, a pattern that was more pronounced in gut tissue (Fig. 1b). *ATP7* is a major copper exporter (Zhou *et al.* 2003), facilitating the transfer of copper from copper accumulating cells in the gut to other tissues. Increased copper export under stressful conditions may play an important role in the response to copper toxicity.

Expression of the copper chaperone *Atox1* was also differentially affected by treatment and tissue. As proteins, Atox1 transports copper to ATP7 (Calap-Quintana *et al.* 2017; Kamiya *et al.* 2018) and to the extracellular antioxidant enzyme SOD3 (Itoh *et al.* 2009; Kamiya *et al.* 2018). Both *Atox1* and *Sod3* were more highly expressed in gut tissue than in head tissue (Fig. 1b), and exposure to copper stress led to a decrease in expression of both genes. Work presented by Itoh *et al.* (2009) suggests that this shift in expression may be mechanistically linked because Atox1 functions both to transfer copper to SOD3 and to regulate its expression as a copper-dependent transcription factor (Itoh *et al.* 2009). Under normal conditions, null mutations in *Atox1* have been shown to lead to copper deficiency, and excess copper led to decreased Atox1 protein expression in wild-type flies (Hua *et al.* 2011).

Overall, we found pervasive differences in expression between tissues, with many genes—including candidate metal responsive genes—showing an expression change in response to copper treatment as well as variation in copper response among tissues. We previously reported a minor effect of DSPR strain-specific copper resistance on the gene expression response to copper (Everman *et al.* 2021). Classifying DSPR strains into copper resistant and susceptible classes in the present study explained relatively little of the expression variation, and resistance class influenced only a small number of genes, none of which are members of known metal response pathways. This finding suggests that the expression data gathered for the 96 DSPR strains examined in the current study does not appear to be clearly associated with resistance measured in Everman *et al.* (2021). This may indicate that the gene expression response may play a modest or even minimal role in copper resistance; however, it is also possible that gene expression patterns underlying differences in copper resistance are detectable in a combination of tissues and/or timepoints that were not assessed in the current study. Equally, effects could be evident at genes with relatively low expression, and the modest read counts we obtained may have been insufficient to properly characterize the expression of these lowly expressed genes.

### Expression QTL mapping

#### Properties of mapped eQTL

eQTL mapping was executed for six datasets: Head-Copper, Head-Control, Head-Response, Gut-Copper, Gut-Control, Gut-Response (see *Methods* for additional detail). In total over all datasets, 4,377 genes were associated with at least one eQTL and 98% of these 4,377 genes were differentially expressed due to one or more of the DE model terms and interactions (described above). For most genes (83–98%; Table 1), we detected one eQTL (either *cis* or *trans*) per gene per dataset (Supplementary Fig. 4). Genes with more than one eQTL per dataset included a multidrug resistance gene *Mdr65* and two cytochrome p450 genes *Cyp6t3* and *Cyp12d1-d*, which have been linked to response to insecticides (Daborn *et al.* 2007; Esteves *et al.* 2021). Two genes linked to copper ion binding and homeostasis (*Mco1* and *CG6908*, Lang *et al.* 2012; Thurmond *et al.* 2019) were also among the genes with multiple eQTL per dataset. However, no gene enrichment related to metal detoxification or response was evident in genes with more than one eQTL peak per dataset. Gene annotation and GO analysis for genes with multiple eQTL is available in the Supplementary data.

**Table 1.** Summary statistics for eQTL mapping analyses.

Analysis	eQTL Type	*N* eQTL Peaks	*N* genes with eQTL	Total *N* unique genes with eQTL	% Genes with 1 eQTL peak	Mean percent variance (range)
Gut-Control	*cis*	1,784	1,779	1,993	96%	43.1 (25.0–74.4)
Gut-Control	*trans*	295	286			33.7 (10.4–63.0)
Gut-Copper	*cis*	1,677	1,674	1,860	97%	42.4 (22.0–76.0)
Gut-Copper	*trans*	248	237			33.4 (21.2–68.2)
Gut-Response	*cis*	139	138	316	98%	40.0 (25.5–72.5)
Gut-Response	*trans*	183	180			32.3 (16.6–41.9)
Head-Control	*cis*	1,875	1869	2,125	96%	43.6 (22.8–76.8)
Head-Control	*trans*	334	320			33.4 (18.3–66.4)
Head-Copper	*cis*	1,879	1,870	2,152	97%	43.6 (23.9–78.1)
Head-Copper	*trans*	351	343			34.2 (20.5–67.4)
Head-Response	*cis*	34	34	197	83%	34.3 (21.3–64.3)
Head-Response	*trans*	235	167			30.0 (18.8–37.5)

For each of the Control and Copper datasets, *cis*-eQTL outnumbered *trans*-eQTL (Table 1). In both Response datasets, we identified fewer eQTL overall but *trans*-eQTL were more common than *cis*-eQTL (Table 1). This pattern is anticipated because the Response datasets are based on the difference in gene expression read counts between control and copper treatments, which should eliminate genetic effects on expression that are consistent in both treatments. Since the bulk of treatment-specific eQTL are *cis*-eQTL, the Response data set is expected to have fewer eQTL with local effects.

Across all datasets, the percent variance in gene expression explained by individual eQTL ranged from 10.4% to 78.1% (Supplementary Fig. 5, Table 1). Consistent with previous work (Dixon *et al.* 2007; Emilsson *et al.* 2008; King *et al.* 2014; Albert *et al.* 2018; Keele *et al.* 2020), *cis*-eQTL tended to have higher percent variance estimates compared to *trans*-eQTL in each of the six datasets (Supplementary Fig. 5, Table 1), suggesting that *cis*-eQTL tend to have a larger effect on transcriptional variation compared to *trans*-eQTL (reviewed in Gibson and Weir 2005; Pierce *et al.* 2014; Võsa *et al.* 2021). However, percent variance estimates are likely overestimated due to Beavis effects so should be assessed with care (Beavis 1995; King and Long 2017).

#### Tissue specificity of mapped eQTL

**Fig. 2.**
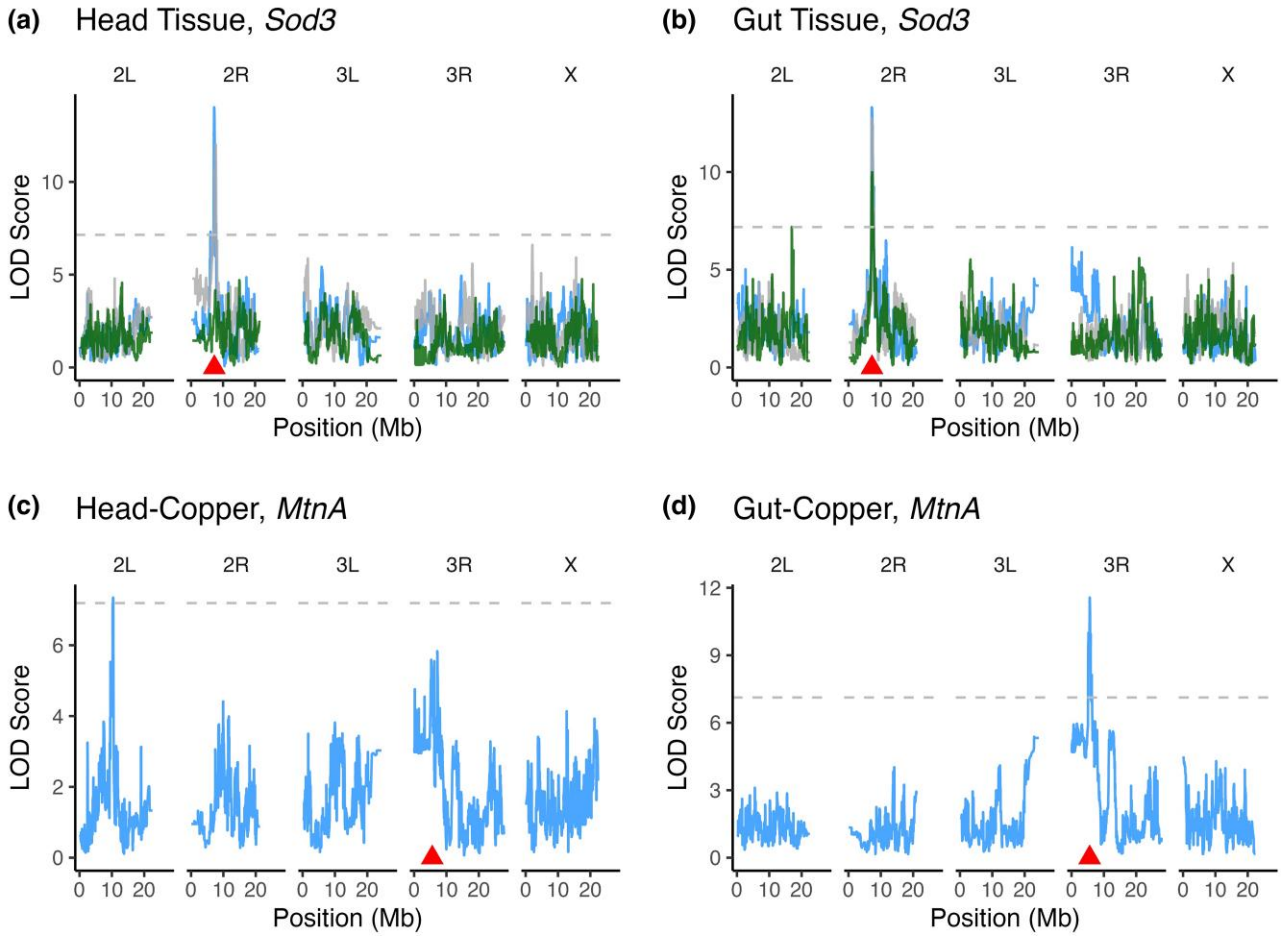
Many genes (53.7%) were associated with eQTL in multiple datasets, but these eQTL were often localized to distinct intervals. a, b) Expression of the gene Sod3 was associated with *cis*-eQTL in all datasets except Head-Response. c, d) The gene MtnA was associated with a *trans*-eQTL on chromosome arm 2L in the Head-Copper dataset (c) and was associated with a *cis*-eQTL in the Gut-Control dataset (d). In all plots, the dashed horizontal line indicates the significance threshold based on permutation and the red triangle point indicates the position of the gene. LOD curves are colored based on treatment: gray = Control, blue = Copper, green = Response.

Although most genes had only one eQTL per dataset (Table 1), 53.7% of genes had one or more eQTL in at least two datasets. For instance, expression of the extracellular copper/zinc superoxide dismutase *Sod3* was associated with a single eQTL per dataset but a similarly located *Sod3 cis*-eQTL was detected in five of the six datasets (Fig. 2a and b). For other genes, eQTL in different datasets were clearly distinct. For example, expression of the metallothionein gene *MtnA* was associated with a *trans*-eQTL in the Head-Copper dataset and a *cis*-eQTL in the Gut-Copper dataset (Fig. 2c and d). This implies that tissue-specific variants influence *MtnA* expression under copper stress.

To determine whether a similarly localized eQTL was detected in multiple datasets, we examined eQTL overlap on a per gene basis between tissues within treatment (Head-Control vs Gut-Control, Head-Copper vs Gut-Copper, Head-Response vs Gut-Response) and within tissue between treatments (Heads-Control vs Heads-Copper and Gut-Control vs Gut-Copper). eQTL were classified as overlapping if the peak positions were within 1.5cM or if the peak intervals overlapped (see *Methods*). Overall, *cis*-eQTL were more likely than *trans*-eQTL to be detected in multiple datasets regardless of which datasets were compared (Supplementary Fig. 6). The number of eQTL that were detected in more than one dataset was highest in within-tissue comparisons (Supplementary Fig. 6a and b), whereas more eQTL were distinct between tissues within treatment (Supplementary Fig. 6c and d). eQTL detected in the Response datasets were most distinct with only seven *cis*-eQTL and no *trans*-eQTL shared between Head- and Gut-Response datasets (Supplementary Fig. 6e). Together, these results are consistent with our DE analysis, which identified tissue as having the greatest impact on expression variation.

Within each tissue the majority of *cis* Response eQTL (64–85%) were also detected in the Control and Copper datasets while the majority of *trans* Response eQTL were unique to the Response dataset (Supplementary Fig. 6f–i). The overlapping *cis* Response eQTL were typically detected as *cis*-eQTL in both Control and Copper datasets, suggesting that these *cis*-eQTL are either associated with different variants in the Control and Copper datasets or that the variant has different additive or magnitude effects on the expression of genes under Control and Copper conditions. Thorough tests of this hypothesis are beyond the scope of our data; however, correlations between Response and Control or Copper founder haplotype effects at each eQTL suggest both patterns may contribute (Supplementary Fig. 7).

Overall, our eQTL mapping results suggest that regulatory variation influences gene expression in a tissue-specific manner for 2,581 genes with distinct eQTL (detected in only one dataset) that were involved in a broad range of processes spanning response to stimulus to cellular metabolism (hypergeometric test for overrepresentation of GO terms, adj *P* < 0.001; Supplementary Fig. 8). In contrast, regulatory variants influenced expression of 705 genes with shared eQTL that were detected in both head and gut tissues following copper exposure (Supplementary Fig. 8). These 705 genes were enriched for a smaller number of GO categories including metal response, detoxification, oxidative stress response, and similar stress response pathways (adj *P* < 0.001; Supplementary Fig. 8). This observation suggests that the control of some major components of metal stress response is potentially consistent across tissues. However, our data provide some evidence that founder haplotype effects at shared eQTL are not always consistent, suggesting that physically overlapping eQTL do not necessarily represent identical genetic effects. For example, founder haplotype effects at shared *cis*-eQTL in Head and Gut tissue associated with *Mdr49*, *Mdr65*, and *Sid* [involved in insecticide resistance (Denecke *et al.* 2017; Sun *et al.* 2017) and oxidative stress response (Seong *et al.* 2014)] were negatively correlated, implying the regulatory variant may have opposite effects on expression of these genes in head and gut tissue, or that the eQTL actually represent the effects of distinct variants (Supplementary Fig. 9).

#### Treatment specificity of eQTL

**Fig. 3.**
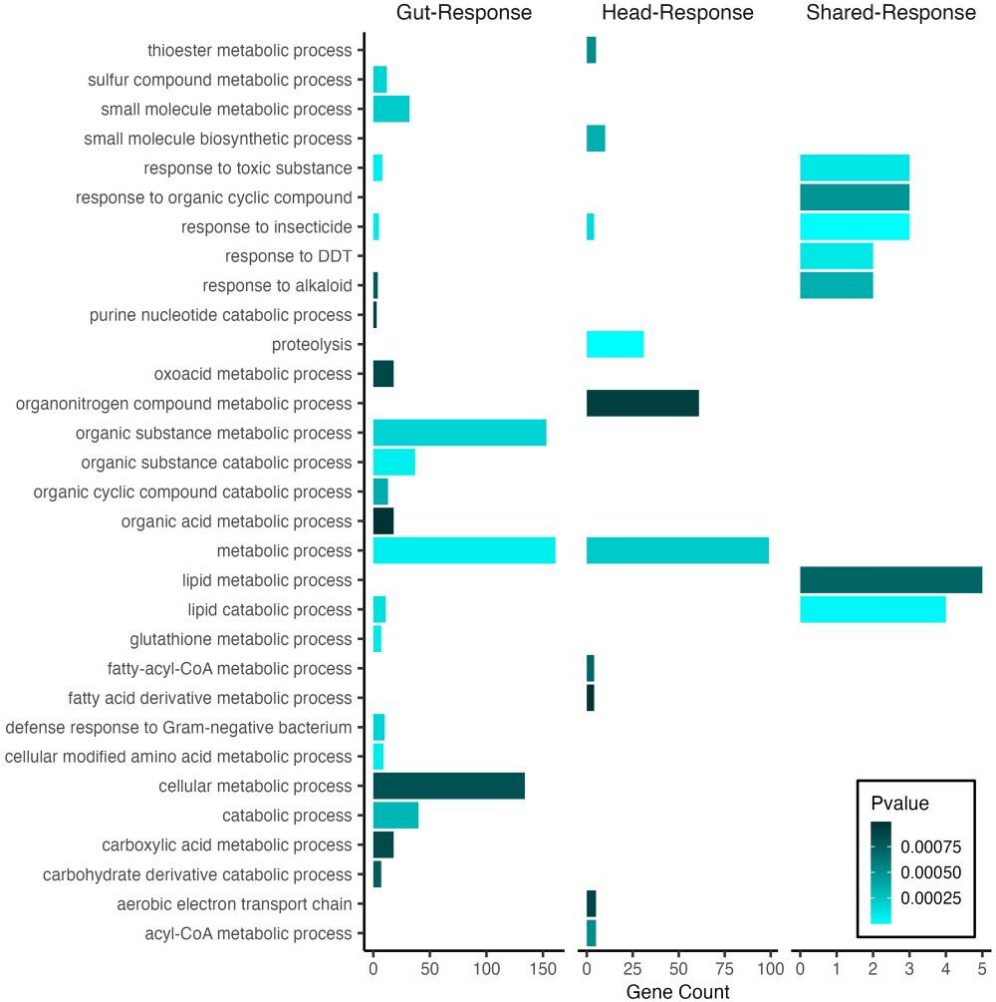
Enrichment of GO categories for Response eQTL genes by tissue (head tissue = 197 genes; gut tissue = 316) and for the set of eQTL genes that were shared between tissues (shared-response, 19 genes).

Treatment-dependent eQTL were identified using the Head- and Gut-Response datasets. Response eQTL reveal loci that impact the difference in gene expression between copper and control conditions in a genotype-specific manner and are thus inherently genotype by environment eQTL. Response eQTL detected in both Gut- and Head-Response datasets were associated with several potential candidate genes that may play a role in the response to copper toxicity. GO analysis of genes with Gut-Response eQTL highlighted categories related to detoxification pathways and response to toxins including glutathione s transferase family genes (glutathione metabolic process; hypergeometric test for overrepresentation of GO terms, adj *P* < 0.001; Fig. 3) and cytochrome p450 genes (response to toxic substances and insecticides, adj *P* < 0.001; Fig. 3) (Kim and Yim 2013; Esteves *et al.* 2021). We also detected Response eQTL for two ABC transporter multidrug resistance protein genes (*Mdr49* and *Mdr69*) as well as two genes that are known to play a role in copper metabolism or binding (*Sod3* and *Tbh*) (Itoh *et al.* 2009; The UniProt Consortium 2021).

Head-Response eQTL included fewer potential candidate genes. Top GO enrichment categories included proteolysis and metabolic process (adj *P* < 0.0001) followed by response to insecticide (adj *P* < 0.001; Fig. 3). We detected Head-Response eQTL for ABC transporter gene *Mdr50* as well as a different *trans*-eQTL associated with *Mco1* that was distinct from those identified by comparing the copper and control-specific head eQTL. Of the 197 annotated Response eQTL genes, 26 play or are predicted to play a role in metal ion binding. There were 19 eQTL genes that were shared between the Head- and Gut-Response datasets, which included several cytochrome p450 family genes. The shared set of Response eQTL were associated with expression of genes that contribute to response to insecticide (adj *P* < 0.0001), response to toxic substance (adj *P* < 0.001) and response to DDT (dichlorodiphenyltrichoroethane) (adj *P* < 0.001) (Fig. 3).

## Discussion

### Copper exposure results in tissue- and treatment-specific expression

The genetic architectures of complex traits such as stress resistance and disease are highly context-dependent and can vary across scales as broad as between species and populations (Whitehead and Crawford 2005; Breschi *et al.* 2016; Findley *et al.* 2021) and as small as between individuals, regions of a particular tissue (Fournier and Schacherer 2017; Çelik and Akdaş 2019; Munro *et al.* 2022), or even within individuals across age or development (Everman and Morgan 2018; Huang *et al.* 2020; Everman *et al.* 2021). Given anticipated tissue-specific gene expression patterns resulting from the spatial distribution of specialized copper-accumulating cells (Calap-Quintana *et al.* 2017; Miguel-Aliaga *et al.* 2018) and the potential for copper toxicity to result in acute damage to digestive tissues as well as neurological tissues (e.g. Tchounwou *et al.* 2008; Jomova *et al.* 2010), one of our principal goals was to characterize the tissue-specific transcriptomic response to copper toxicity. We demonstrate striking patterns of tissue- and treatment-specific genetic response to copper exposure using a combination of DE analysis and eQTL mapping. More than 90% of all genes had tissue-specific patterns of expression, and a significant tissue by treatment interaction affected 70% of the genome. Our eQTL mapping results provide similar insight; the majority of eQTL were tissue-specific within treatment (Supplementary Fig. 6), suggesting that the genetic control of gene expression response to stress is highly context-dependent.

In general, overexposure to heavy metals results in the accumulation of reactive oxygen species (ROS) and activation of oxidative stress response pathways (e.g. Ercal *et al.* 2001; Gaetke 2003; Uriu-Adams and Keen 2005). Especially for heavy metals that are biologically necessary, there are also metal-specific metabolism pathways that can contribute to the metal toxicity response (Calap-Quintana *et al.* 2017). In the case of copper, specialized cells that are involved in uptake, metabolism, and detoxification of copper ions from the diet line the middle midgut of the fly and the acidic region of the digestive system in vertebrates (Dubreuil 2004; Calap-Quintana *et al.* 2017). Many of the genes that have been linked to copper response in previous studies were significantly differentially expressed under copper conditions relative to control conditions in our study (Fig. 1), and our eQTL mapping results suggest that many of these copper-responsive genes are influenced by genetic variation in regulatory elements. Furthermore, we found that genes with functions related to metal response followed expected tissue-specific expression patterns (e.g. *Mtn* family genes; *Ctr1A* and *Ctr1B*; *Atox1* and *SOD3*; *ATP7*; discussed above (Zhou *et al.* 2003; Turski and Thiele 2007; Itoh *et al.* 2009; Calap-Quintana *et al.* 2017; Kamiya *et al.* 2018)) (Fig. 1). For instance, and consistent with previous reports, we found that *MtnA* expression was strongly induced by copper exposure (Fig. 1b); however, the level of induction was much more pronounced in gut tissue compared with head tissue, leading to a significant tissue by treatment interaction. We also found variation in the genetic control of *MtnA* expression under copper conditions that followed a tissue specific pattern. In gut tissue, *MtnA* expression in response to copper is influenced by a *cis*-eQTL (Fig. 2c), whereas in head tissue *MtnA* expression under copper conditions is influenced by a *trans*-eQTL (Fig. 2d). Although the genetic variants that contribute to *MtnA* expression in either tissue are present in both tissues, each variant appears to only influence gene expression in a particular tissue.

While *D. melanogaster* is commonly used to characterize the genetic control of heavy metal response (e.g. Egli *et al.* 2003, Egli, Yepsikoposyan *et al*. 2006; Balamurugan *et al.* 2004; Norgate *et al.* 2010; Hua *et al.* 2011), tissue-specific comparisons are not common (Fasae and Abolaji 2022). One of the few examples of tissue-specific response to heavy metal toxicity in *D. melanogaster* demonstrated that variation in *CncC* pathway activity (involved in autophagy regulation) across muscle and neurological tissue contributed to the toxicity response to methylmercury (Gunderson *et al.* 2020). Our DE analysis provides novel insight and suggests that there are tissue-specific patterns in broad responses to stress through the differential activation of pathways. Although all the stress response categories represented by our subset of genes can be linked to heavy metal stress response (Kefaloyianni *et al.* 2005; Darling and Cook 2014), our data suggest that oxidative stress response is the primary stress response mechanism to copper exposure in neurological tissue (proxied with head tissue in our study) while the primary stress response mechanisms in gut tissue are more diverse. Because previous studies that offer insight into tissue-specific gene expression response often focus on sets of tissues that infrequently overlap, it is challenging to determine whether the patterns we observe in our study are unique or reflective of a general pattern. However, our data do strongly suggest that there is a tissue-specific response to copper toxicity over the 8-hour exposure period used in this study. Whether this difference is due to tissue-specific vulnerability, to mode of exposure, to temporal progression of copper ions throughout the organism, or a combination of these and other factors remains to be fully determined. Additional follow up with tissue-specific knockdown or ablation of key genes involved in oxidative stress and other stress response pathways would be needed to strengthen these observations.

### Transcriptomic response to copper stress is influenced by regulatory allelic variation

Allelic variation and its contribution to trait variation has been repeatedly dissected, characterized, and functionally tested in the context of complex traits ranging from those associated with human disease (e.g. Granhall *et al.* 2006; Hardy *et al.* 2018) to various stress responses including heavy metal resistance (e.g. Zhou *et al.* 2017; Evans *et al.* 2018; Everman *et al.* 2019, 2021). In addition to the potential to influence the functional gene product, allelic variation can also influence the response to stress via effects on gene expression patterns. The majority of allelic variation is found in noncoding regions of the genome (Umans *et al.* 2021), leading to the expectation that these noncoding variants play a regulatory role. By treating expression of individual genes as traits measured in multiple treatments and tissues, we were able to gain insight into context-dependent genetic control of gene expression using eQTL mapping. Approximately half of the annotated *Drosophila* genome was associated with one or more eQTL in our study, providing ample evidence of genetic variation in the control of gene expression (Table 1). Nearly all (98%) of genes that were associated with at least one eQTL were also differentially expressed, suggesting that the variants associated with these genes likely influence expression, although functional validation would be necessary to test this hypothesis. This observation is consistent with work by Boyle *et al.* (2017) demonstrating that SNPs which contribute to trait variation are enriched near genes that are actively transcribed under disease states; our observations suggest this pattern extends to stress conditions as well.

Although eQTL mapping provides detailed insight into the genetic control of a given trait by examining elements that contribute to trait variation on a gene-by-gene basis, there are several challenges with this approach. Power to detect eQTL with modest effects is directly related to the number of strains in which the trait is measured (Ruden *et al.* 2009; King, Macdonald *et al*. 2012; Qu *et al.* 2018; Arvanitis *et al.* 2022). By using 93–96 strains in our eQTL analyses, lack of power to detect modest-effect eQTL likely contributes to our estimates of tissue-specificity. For example, power estimates to detect a QTL with an effect size of 10 with 100 DSPR strains is approximately 20% (King, Macdonald *et al*. 2012). Detection of tissue-specific eQTL is also more sensitive to false negatives, for example due to low levels of expression of a particular gene in a given tissue. We also note the increased likelihood of detecting false positives given the large number of tests completed for six datasets. However, despite these challenges our data suggest that allelic variation that contributes to the regulation of gene expression is tissue-specific for approximately 65% of eQTL detected in the control and copper datasets.

Similar studies of tissue-specific eQTL mapping are relatively rare in model organisms but have provided examples of tissue-specific eQTL. Using a mouse multiparental mapping population (Collaborative Cross, Aylor *et al.* 2011) that is similar in concept to the DSPR, Keele *et al.* (2020) demonstrated that among lung, liver, and kidney tissue genetic control of several genes varied across tissues. Spatially discrete genetic control of gene expression can also vary at the subtissue level. Munro *et al.* (2022) demonstrated gene expression patterns that are specific to five regions of the rat brain are influenced by a combination of shared and subtissue-specific eQTL. Shared eQTL were most common in similar brain tissue types, but subtissue-specific eQTL were common between more distinct types of brain tissue (Munro *et al.* 2022). eQTL mapping studies of human disease and native state gene expression have also provided evidence of tissue- and cell type-specific genetic control of gene expression (Dimas *et al.* 2009; Nica *et al.* 2011; Fairfax *et al.* 2012; Peters *et al.* 2016; GTEx Consortium 2017; Gamazon *et al.* 2018). A recent study presenting a novel analytical approach to identify colocalized eQTL suggests that tissue-specific eQTL may be more common than previously thought and key for identifying regulatory variants associated with complex disease traits (Arvanitis *et al.* 2022). The existence of other examples of tissue-specific eQTL combined with our evidence strongly suggest that tissue-specific eQTL contribute to the stress response to copper toxicity in *D. melanogaster*.

### Consistent vs tissue-specific effects of eQTL

In addition to identifying tissue-specific eQTL, our study also provides insight into eQTL that have consistent vs environment-dependent effects within tissue type. Complex traits are dependent on both genotype and environmental variation, and the interaction between genotype and environment can contribute to overall susceptibility of individuals to stress and disease (Li *et al.* 2006; Duveau and Félix 2012; Moyerbrailean *et al.* 2016; Lea *et al.* 2022). The importance of genotype by environment interactions and the detection of quantitative loci with context-dependent effects was demonstrated by Lea *et al*. (2022) in a study using 544 human cell lines exposed to 12 environments. Using a high-powered experimental design, they demonstrated that the complex traits measured were influenced by a combination of eQTL with consistent effects in multiple environments as well as eQTL with environment-dependent effects (Lea *et al.* 2022). Similarly, Umans *et al*. (2021) offer a review and perspective on the importance of examining regulatory variants in multiple contexts. Recognizing the dynamic nature of context-dependent effects of alleles associated with disease in humans, Umans *et al.* (2021) propose that a critical approach to characterizing allelic variation in regulatory elements is to use multiple treatment conditions to detect important disease associated variants.

Overall, the majority of the eQTL we detected were near the position of the gene they were associated with (*cis*-eQTL), suggesting that genetic variation in local regulatory elements plays an important role in the gene expression response to copper stress. *cis*-eQTL generally have larger effects on trait variation and are thus easier to detect with modestly powered designs (Hill *et al.* 2021), and *trans*-eQTL are additionally difficult to detect because they may not act at the level of mRNA regulation but instead have larger detectable effects at the protein level (Boyle *et al.* 2017). *cis* regulatory variation is widely appreciated to be an important contributor to phenotypic variation and plays a decisive role in the evolution of traits in natural populations and in human disease (Wray 2007; Savinkova *et al.* 2009; Wittkopp and Kalay 2011; Hill *et al.* 2021). At the treatment level and consistent with previous studies in humans, flies, and worms (Ruden *et al.* 2009; Snoek *et al.* 2017; Qu *et al.* 2018; Sterken *et al.* 2020; Lea *et al.* 2022), we found eQTL consistently in both treatments as well as eQTL that were only detected in one of the two treatments (Supplementary Fig. 6). The number of eQTL that were consistently detected was higher than treatment-specific eQTL, similar to a pattern previously reported for human cell lines (Lea *et al.* 2022). Similarly, in *D. melanogaster*, Qu *et al.* (2018) and Ruden *et al.* (2009) demonstrated that a small but non-negligeable number of eQTL were only detected following exposure to lead in head tissue or whole animals, respectively. While our results highlight the potential for even eQTL that are repeatedly detected across treatments to have treatment-specific effects on the expression of a given gene, adding a deeper dimension to gene by environment interaction eQTL, it is important to consider that the QTL intervals may include more than one variant that influences gene expression. One of these variants may influence expression under control conditions while the other influenced expression under copper conditions. Without additional follow-up studies, our analyses do not provide sufficient resolution to distinguish this case from one in which the same variant has treatment-specific effects on gene expression.

In addition to characterizing genotype by environment eQTL by examining the difference between the control and copper datasets for each tissue, we also examined eQTL that were associated with a summary metric of the gene expression response to copper stress. The majority of eQTL detected in the Head- and Gut-Response datasets were *trans-*eQTL. Our study design partially accounts for the deficit in *cis*-eQTL in the Response datasets, as any effects of *cis*-eQTL that were detected with similar effects in control and copper datasets would be reduced in the Response eQTL mapping analyses. However, an enrichment of *trans*-eQTL associated with specific environments has been previously observed in *C. elegans* in response to temperature stress (Li *et al.* 2006; Snoek *et al.* 2017). *trans*-eQTL that are associated with the dynamic shift in gene expression response to the environment call attention to allelic variants that may play a role in the regulation of stress-dependent expression change that may otherwise not be detected if the treatment conditions are considered independently. We found several Response *trans*-eQTL near genes with a wide range of functions related to response to toxic conditions (Fig. 3) that highlight potential candidate regulatory QTL. Additional follow up would be necessary to corroborate our observations and to fully characterize the role that these potential QTL sites may play in the regulation of the response to copper stress.

### Correspondence between eQTL and previous work

Regulatory variation has been established as an important contributor to trait variation in diverse species and for diverse traits with evolutionary and biomedical consequences (reviewed in Wray 2007; Pai *et al.* 2015). SNPs associated with variation in gene expression can modify phenotypic variation through transcriptional and translational mechanisms (reviewed in Pai *et al.* 2015), and regulatory SNPs can have multiple effects, for example modifying histones that influence epigenetic regulation as well as impacting the binding of transcriptional machinery that directly changes mRNA levels (Rockman and Kruglyak 2006; Duncan *et al.* 2014; Pai *et al.* 2015). While traditional phenotype GWAS and QTL mapping studies can provide important insight into the genetic architecture of complex traits, the candidate SNPs frequently fall in noncoding regions and candidate genomic regions implicated by these studies are often not narrow enough to clearly definitively identify the specific variants that influence trait variation. eQTL mapping studies of traits that have also been characterized using GWAS or QTL mapping can assist with refinement and characterization of variants that influence trait variation by examining overlap between studies (Nica *et al.* 2010; Nica and Dermitzakis 2013; Pai *et al.* 2015). We previously examined phenotypic variation in copper resistance in a larger set of DSPR strains and identified six QTL (referred to herein as pQTL) that encompassed 1,369 protein coding genes, a fraction of which may contribute to variation in copper resistance in the B panel of the DSPR (Supplementary Table 4 of Everman *et al.* 2021). While not all of the genes under the six pQTL peaks will contribute to copper resistance, overlap between pQTL genes and eQTL may help further characterize the genetic control of copper resistance.

*cis-*eQTL that are associated with genes that fall under pQTL intervals may be more likely to contribute to regulatory variation that influences copper resistance (Pai *et al.* 2015). Of the 1,369 pQTL-associated genes, 906–910 were tested for eQTL. *cis-*eQTL were detected for between 0.4% and 24% of the pQTL associated genes (Supplementary Table 3). In our previous study, we explored the contribution of 16 potential candidate genes to copper resistance using RNAi knockdown and found that all but six influenced copper resistance (Supplementary Fig. 10 of Everman *et al.* 2021). The majority of these previously identified candidate genes also had *cis-*eQTL in this study (Supplementary Table 4); however, these *cis-*eQTL were frequently detected under both control and copper conditions, potentially reducing their likelihood to be strong candidates. Additional tests would be necessary to validate the potential mechanistic link between genes identified in our pQTL and eQTL mapping studies.

We also determined the number of *trans-*eQTL that overlapped with pQTL regions, using peak positions of *trans*-eQTL and the genomic intervals defined for each pQTL in our previous paper (Supplementary Table 1 in Everman *et al.* 2021). Between 2 and 57 *trans*-eQTL fell within each pQTL interval. For all but one gene (*CG30357*) with overlapping *trans*-eQTL, *trans*-eQTL genes and pQTL interval genes were distinct. Two genes with overlapping *trans*-eQTL (*GstO1* and *Zip42C-1*) have been previously linked to detoxification (Gramates *et al.* 2022; Martin *et al.* 2023), and seven genes with overlapping *trans*-eQTL have been linked to oxidative or endoplasmic stress (*Edem2*, *CG15547*, *Khc*, *rl*, *shep*, *slim*, and *IP3K1*) (Gramates *et al.* 2022; Martin *et al.* 2023). None of the genes with *trans*-eQTL within the pQTL regions have been previously linked to copper resistance, response, or toxicity.

Other studies have noted modest overlap between pQTL and eQTL mapping studies (e.g. Steibel *et al.* 2011; Van Den Berg *et al.* 2019), pointing to lack of power to detect QTL (particularly *trans*-eQTL; Nica *et al.* 2010; Pai *et al.* 2015) and key differences in the compared studies related to the tissues assessed. Because our previous work examined phenotypic variation in copper resistance exhibited by whole adult females and the current study examined expression variation in specific tissues in response to copper stress in a subset of the previously assessed DSPR strains, additional follow up would be needed to determine how candidates identified from our pQTL and eQTL mapping studies are related.

## Conclusions

Exposure to heavy metal stress results in a profound change in transcriptional activity across multiple tissues, and the genetic control of the gene expression response varies depending on the environmental conditions and tissue. By taking a combined approach of DE analysis and eQTL mapping, we were able to dissect and characterize more subtle differences in tissue-specific response to copper toxicity. Our work provides a novel set of candidate loci that may have context-dependent effects on gene expression and plasticity. From the patterns that differentiate the genetic response observed in head and gut tissue, we gain deeper insight into the level of toxicity response that is activated by short exposure to copper stress.

## Supplementary Material

jkae015_Supplementary_Data

## Data Availability

RNA-seq reads are available from NCBI SRA BioProject PRJNA993605. All data including DE results, quantile-normalized expression data used for eQTL analyses, and code used to perform eQTL analyses are available from figshare (doi:10.25387/g3.24579133). Supplementary material contains Supplementary Methods that provide more detail on the isolation of head tissue and PCA correction of gene expression in preparation for eQTL mapping. Supplementary material also includes documentation and code used in the alignment pipeline, code to carry out eQTL mapping analysis, and all input and output data generated for each analytical step including raw expression values, complete DE results, and datafiles generated for each eQTL analysis. Supplemental material available at G3 online.
