# Supplementary material for: Gene expression variation underlying tissue-specific responses to copper stress in Drosophila melanogaster
Source: G3 (Bethesda). 2024 Jan 23;14(3):jkae015. doi: 10.1093/g3journal/jkae015 (PMC11021028; doi:10.1093/g3journal/jkae015)
Supplement: jkae015_Supplementary_Data [file jkae015_supplementary_data.zip › Supplemental_Legends_G3-2023-404710.docx]

# Supplemental Figures

**Figure S1.** Adult copper resistance of B panel strains used in the current study. We randomly sampled 48 resistant and 48 sensitive DSPR B panel strains from the top and bottom 25% of the distribution of adult copper survival response following 48-hour exposure to 50mM CuSO_4_ reported in (Everman et al. 2021).

**Figure S2.** Distributions of raw read pair counts by library pool. Each tissue-specific pool included copper and control sample pairs, and an even representation of resistant and sensitive strains. HO stands for “high-output” NextSeq500 flowcell, MO stands for “mid-output” flowcell. Raw reads from the two sequencing runs of the 96-plex Heads library (green shading) were combined prior to read processing.

**Figure S3.** Comparisons of the number of raw read pairs, the number retained following trimming, the percentage aligned, and the percentage assigned to genes following the HISAT2 pipeline from the high-output (HO) and mid-output (MO) sequencing runs for the 96-plex Head library. Apart from raw pair count, which varies due to under-clustering of the 96-plex Head library, data are consistent between the HO and MO sequencing of the same 96-plex library and are comparable to the separate 94-plex Head library that was only sequenced on one HO flowcell.

**Figure S4.** For most genes (>80%), mapping yielded a single eQTL. Data shown are counts of distinct eQTL peaks per gene for each of the 6 datasets. Gut tissue is shown in orange, head tissue is shown in red. Numbers above bars provide the actual number of genes in each category.

**Figure S5.** Percent variance explained by mapped *cis* and *trans* eQTL. *cis* eQTL (blue) tended to have higher estimates compared to *trans* eQTL (red). Estimates were slightly lower in both response datasets for both *cis* and *trans* eQTL. Numbers at the base of each plot report the total number of *cis* and *trans* eQTL detected per dataset.

**Figure S6.** Many eQTL were detected in multiple datasets. Overlap was highest within tissue between treatments (A and B) but was still substantial in comparisons between tissues within treatment (C, D). Comparisons between the Head- and Gut-Response datasets revealed that most eQTL were distinct between the tissue-specific characterization of copper response. Overlap was also high in comparisons of the Response datasets against the tissue- and treatment-specific datasets (F – I). The majority (64 – 81%) of *cis* Response eQTL were shared with either the Control or Copper datasets for the corresponding tissue. For Head tissue, all but 9 *cis* Response eQTL were shared amongst Head-Control or Head-Copper *cis* eQTL; for gut tissue, all but 14 *cis* Response eQTL were shared amongst Gut-Control or Gut-Copper cis eQTL (data not shown).

**Figure S7.** Founder haplotype effects at *cis* eQTL peak positions that were detected in Response, Copper, and Control datasets for a given tissue ranged in strength of correlation for each comparison: A. Head-Response vs Head-Control, B. Head-Response vs Head-Copper, C. Gut-Response vs Gut-Control, D. Gut-Response vs Gut-Copper. Founder effect estimates are impacted by low replication per DSPR strain and should be interpreted with care; however, patterns suggest the detection of Response eQTL in our study may be influenced by a combination of genetic variants with different magnitude effects and treatment-specific additive effects.

**Figure S8.** Enrichment of GO categories for genes with eQTL in Head-Copper, Gut-Copper, or both datasets.

**Figure S9.** Correlations of estimated founder haplotype effects at peak positions of eQTL detected in both the Head-Copper and Gut-Copper datasets skewed positive for the majority of eQTL. A. Estimated founder haplotype effect correlations at eQTL associated with genes with functions unrelated to metal response based on FlyBase (Gramates *et al.* 2022) annotations were generally continuous and ranged from a small number of strong negative correlations to strong positive correlations. B. Estimated founder haplotype effect correlations at eQTL associated with genes with functions that are related to metal response (oxidative stress response, response to copper, response to xenobiotics, detoxification) generally fell into two groups with a small number of strong negative correlations and a larger number of strong positive correlations.

# Supplemental Tables

**Table S1.** Samples from head and gut tissue were arrayed across four 96-well plates. Sample names are written as “AB_00000_CC” where “A” is tissue (H = head, G = gut), “B” is the level of resistance exhibited by the strain in Everman et al. (2021) (R = resistant, S = susceptible), “00000” is the five digit strain ID and “CC” is the treatment (Cu = exposed to copper sulfate, NA = water control). Plates 1 and 2 contained only head samples; plates 3 and 4 contained only gut samples. Copper and control-treated strain pairs were kept together on tissue-specific plates. Each plate had an even representation of resistant and sensitive strains. Copper samples (Cu) are shaded in blue; control samples (NA) are not shaded. Black text indicates resistant strains; red text indicates sensitive strains. Samples for which library prep failed are indicated with strikethrough text (HR_21180_Cu, HR_21180_NA, GR_21180_Cu, GR_21232_NA, GS_21281_NA).

**Table S2.** Principal components used in correction of datasets in preparation for eQTL mapping.

**Table S3.** Overlap between genes previously associated with copper resistance QTL and eQTL detected in this study.

**Table S4.** Previously tested candidate genes with eQTL in this study.
