# Supplementary material for: Gene expression variation underlying tissue-specific responses to copper stress in Drosophila melanogaster
Source: G3 (Bethesda). 2024 Jan 23;14(3):jkae015. doi: 10.1093/g3journal/jkae015 (PMC11021028; doi:10.1093/g3journal/jkae015)
Supplement: jkae015_Supplementary_Data [file jkae015_supplementary_data.zip › Supplemental_Methods_G3-2023-404710.docx]

## Comparison between alignment pipelines

A 3-inch diameter brass sieve with #40 mesh (425 μm, Dual Manufacturing Co, Inc US3-40B) was stacked on a solid base (SP3-1), followed by a sieve with larger #25 mesh (710μm, US3-25B), followed by a cover (SC-3; Figure 1). Frozen flies (held on dry ice to prevent thawing) were dumped into the top 710μm chilled sieve, and the entire stack was vortexed for 30 seconds. The top 710μm sieve caught the headless fly bodies and allowed the heads to be collected in the 425μm sieve, while smaller fly parts fell through the 425μm sieve to the base (Figure 1). The sieve containing heads was quickly tapped onto a funnel directed into a labeled screw top microcentrifuge tube containing 3-4 glass beads. We then added 300uL TRIzol Reagent (Invitrogen, 15596018) to the tube. The heads were kept frozen throughout processing of several samples by storing the microcentrifuge tubes in heat block inserts that were chilled by and surrounded with dry ice. After each sample, the sieves were submerged in E-Pure water, followed by cleaning solution (0.1M NaOH, 1mM EDTA), followed by 95% ethanol. The ethanol was burned off the sieve components to ensure any remaining biological material was incinerated. Once the sieves were cooled, they were once again chilled on dry ice. Funnels were similarly meticulously cleaned between each strain and rinsed with ethanol.


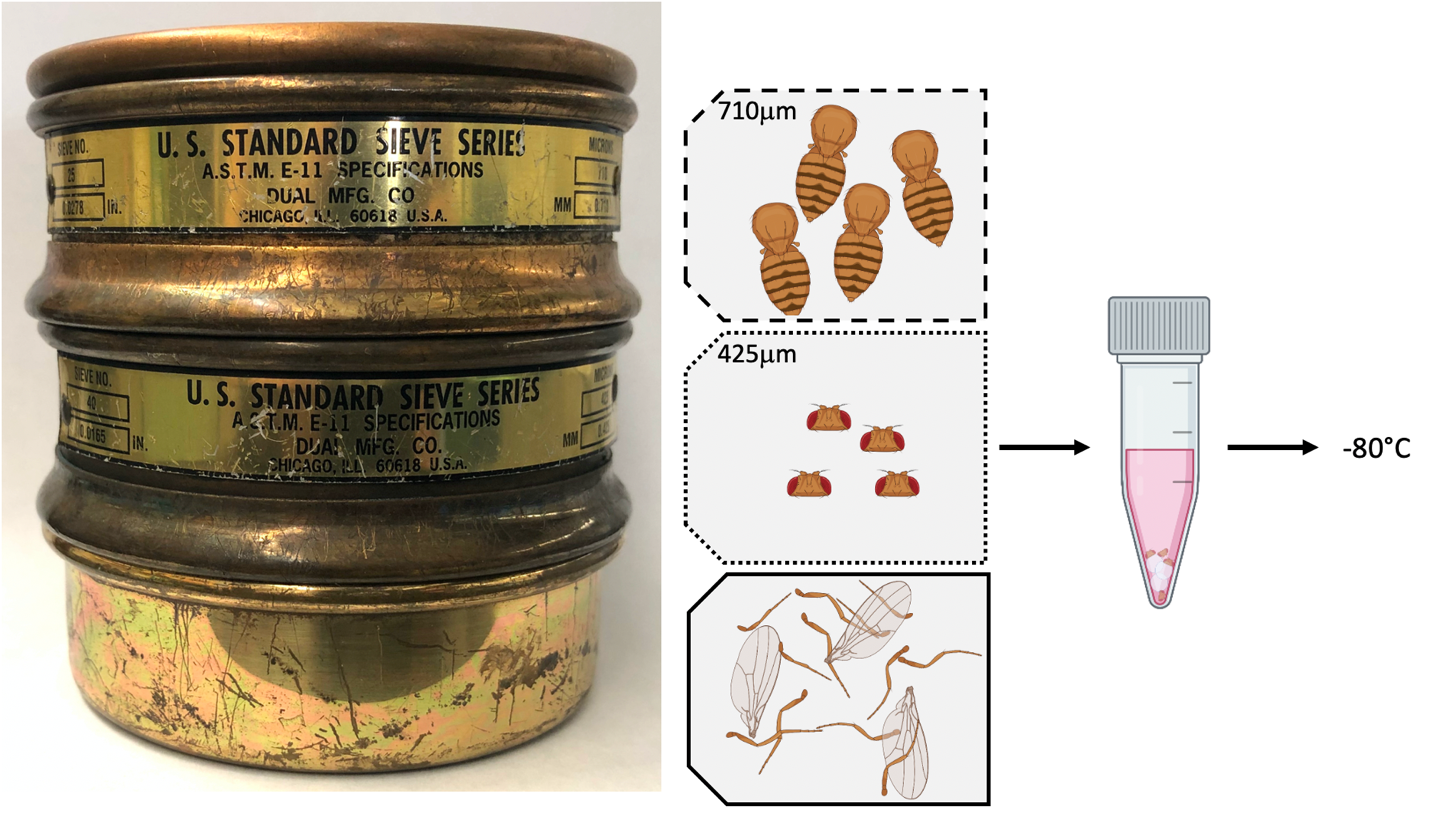


Figure 1. A series of sieves with decreasing mesh size were used to collect heads. Vortexed, frozen flies were dumped into the top sieve, which collected bodies and allowed heads to fall to the second sieve. The second sieve retained the heads and allowed legs and wings to fall to the collection pan. Heads were dumped into screw top microcentrifuge tubes containing glass homogenization beads and held in TRIzol reagent at -80°C until RNA extraction. Created with BioRender.com.

## Comparison between alignment pipelines

We also aligned fastp-filtered reads using the kallisto pseudoalignment pipeline and the Ensembl *Drosophila* transcriptome release 90. Average library size was 3.96 million reads following kallisto alignment. We applied the same post-alignment filtering parameters described above to the kallisto generated counts calculated as the sum of transcripts. To compare gene expression results from the HISAT2 and kallisto pipelines, we calculated gene-wise correlations in expression following log2 transformation and quantile normalization across the 379 libraries. Sample and tissue-specific read counts for the 9434 genes retained in both the kallisto and HISAT2/SAMtools/featureCounts pipeline were highly correlated (mean R = 98.2%, Figure 2). The 267 genes with counts that were less than 90% correlated between the two pipelines were not enriched for any functional or molecular categories following gene ontology analysis (Flymine, (Lyne *et al.* 2007)). Given the high correlation between pipelines, all downstream differential expression (DE) analyses and eQTL mapping presented are derived from the HISAT2/SAMtools/featureCounts pipeline.


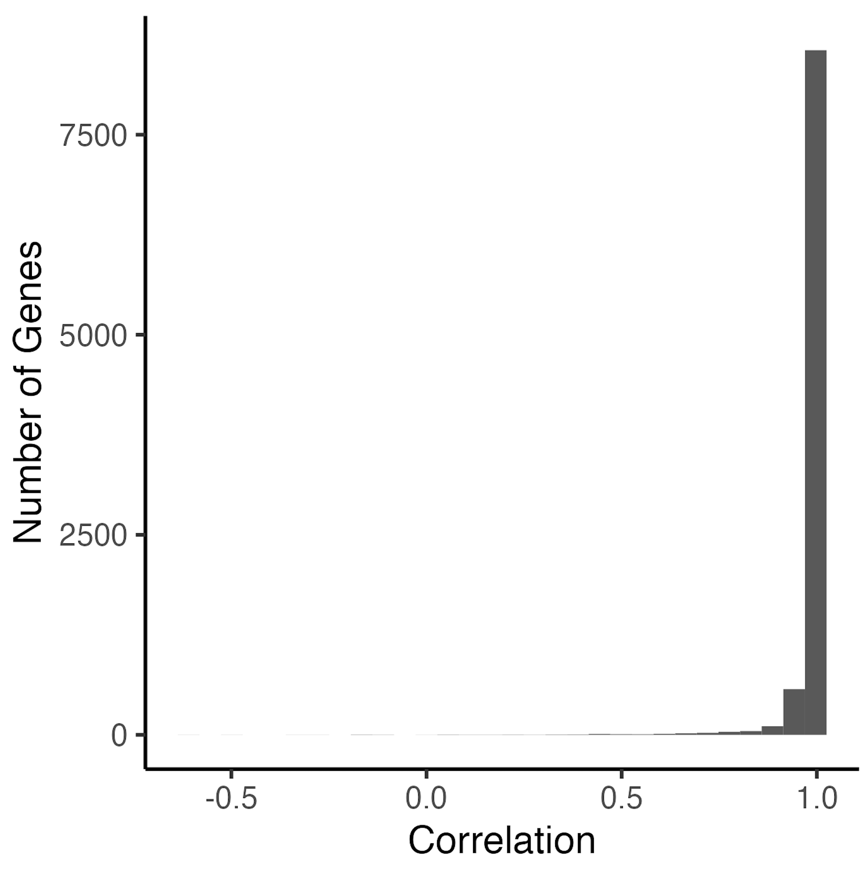


**Figure 2.** Gene expression estimates from the HISAT2 and kallisto pipelines were consistent. After alignment and filtering out of lowly-expressed genes, expression estimates for 9842 and 9913 genes from the HISAT2 and kallisto pipelines, respectively, were retained. Gene-wise correlations in read counts across all samples for shared retained genes (9434) were highly correlated (mean R = 98.2%).

## PCA correction of gene expression for eQTL analysis

A common strategy to reduce spurious gene-eQTL associations in high-dimensional data is to use PCA to account for known and unknown technical or environmental factors (Leek and Storey 2007; Pickrell *et al.* 2010; Gaffney *et al.* 2012; King *et al.* 2014). To determine the most appropriate PCA correction for our data, we compared three versions of the eQTL mapping analysis following quantile normalization using 1) uncorrected data, 2) data corrected for PCs that explained more than 2% of the variance and/or were correlated with tissue collection batch or sample pool (general linear model test of the effect of technical variation on PC, uncorrected α = 0.05) and 3) data corrected for PCs that explained more than 1% of variance and/or were correlated with technical variation. The set of PCs accounted for in each case are listed in Table S2. For brevity, we will simply refer to the two PCA-corrected versions of the dataset as 2% and 1% corrected. Corrections were made by regressing out PCs separately for each of the six expression datasets. Residuals were quantile normalized once more and treated as gene expression values for each of the eQTL analyses and for all downstream analyses.

We performed the three eQTL analyses (no correction, 2% correction, 1% correction) for each of the six datasets as described above. In Head-Control, Head-Copper, Gut-Control, and Gut-Copper eQTL analyses, failing to correct for technical variation via PCA resulted in many more peaks far from the target gene compared to the 2% and 1% cutoffs. Because not correcting for technical factors increases noise, it is likely the large number of distant peaks in the uncorrected data includes many more false positives than true *trans* eQTL (Figure 3). The relationship between peak number and data correction strategy was more pronounced for head samples, likely due to the known technical effect of sequencing pool (Figure 3) that we account for in the PCA-corrected versions. Head- and Gut-Response data were less sensitive to the PCA correction (Figure 3, 4). Removing PCs explaining 1% of the variance further reduced the total number of peaks in both head and gut analyses relative to the 2% variance correction (Figure 4). However, this difference was slight, and overall levels of noise observed in individual LOD plots were visually and statistically similar between the 2% and 1% data correction (average correlation in genome-wide LOD scores between 1% and 2% corrected data for genes with eQTL ranged from 70% - 83%; Figure 5). To avoid removing true positives through use of a strict 1% cutoff, all subsequent analyses/results employ the 2% PCA-corrected version of the dataset.


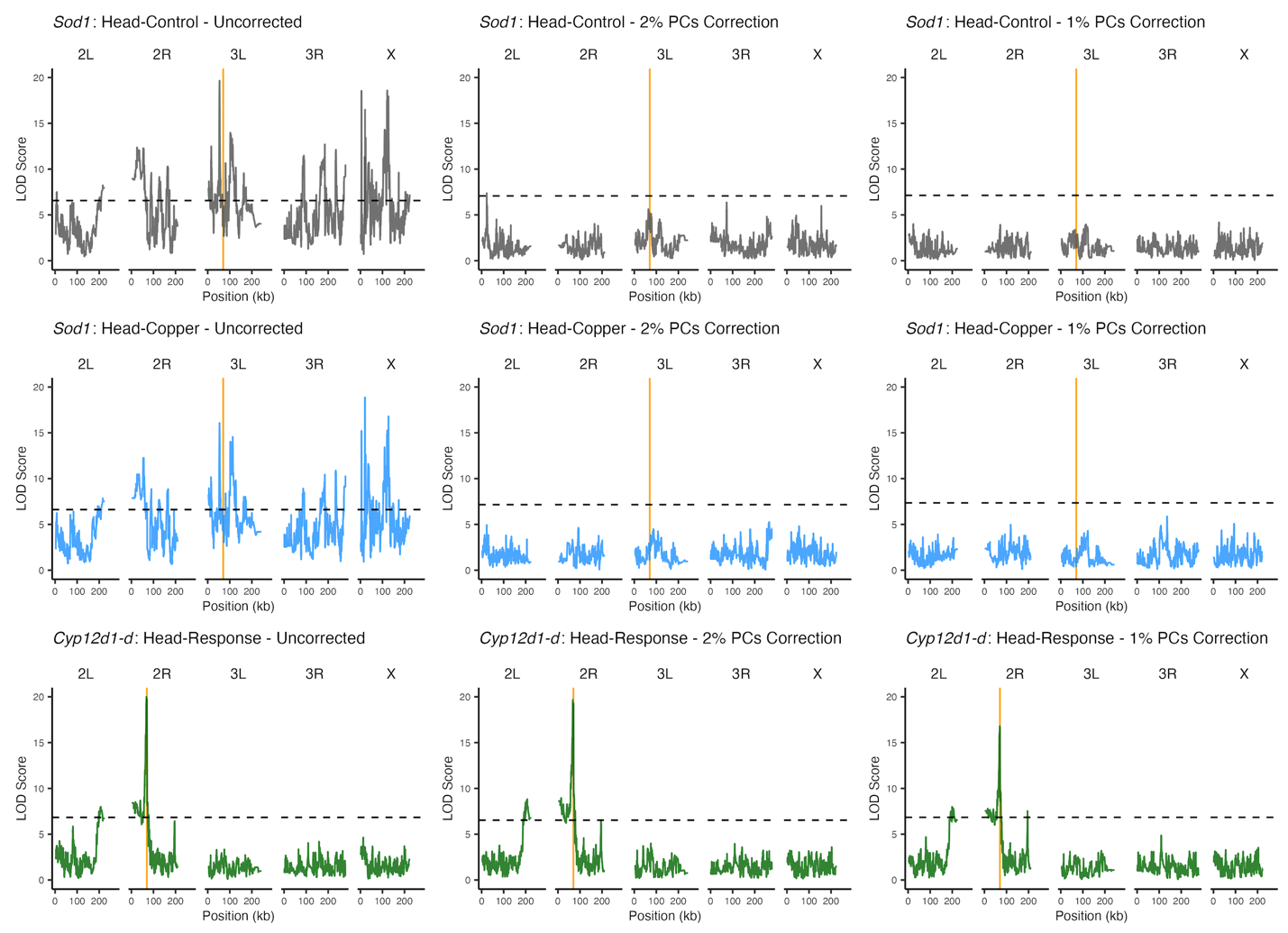


**Figure 3.** Correcting for known and unknown sources of technical variation reduced noise in eQTL mapping. Head-Control (top row) and Head-Copper (middle row) eQTL maps for *Sod1* are noisy when technical variation is not accounted for (left column). Correcting for 2% (middle column) and 1% PCs (right column) along with those associated with known technical factors appears to limit noise. Head-Response data (bottom row) were less sensitive to data correction. In each plot, orange vertical lines indicate the location of the gene. Horizontal lines indicate the gene- and dataset-specific significance threshold.


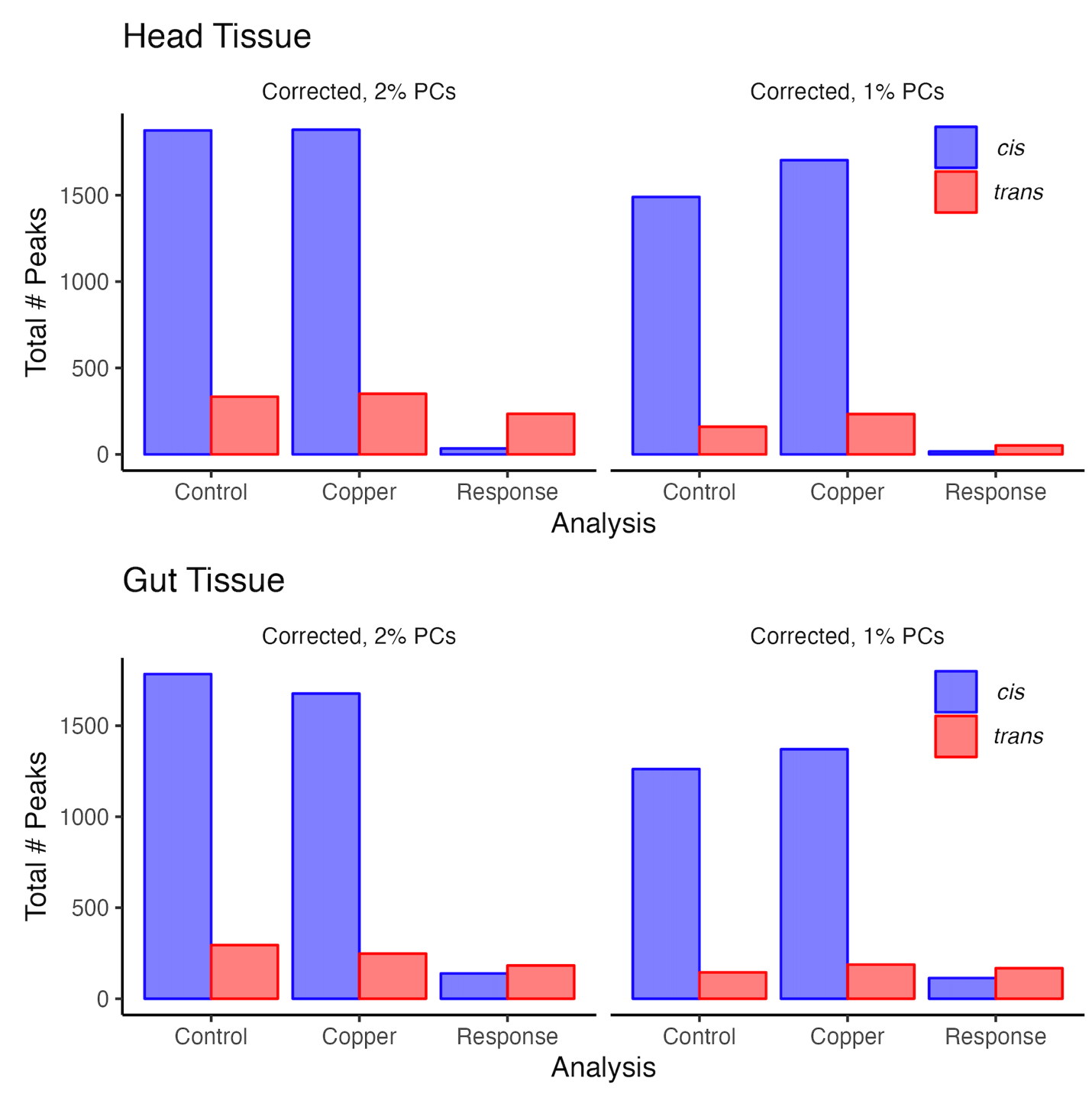


**Figure 4.** Correcting for PCs that explained 2% or 1% of the variation in gene expression resulted in similar numbers of eQTL. Head samples are shown in A; Gut samples are shown in B. In both panels, the left column shows the 2% correction, the right column shows the 1% correction.


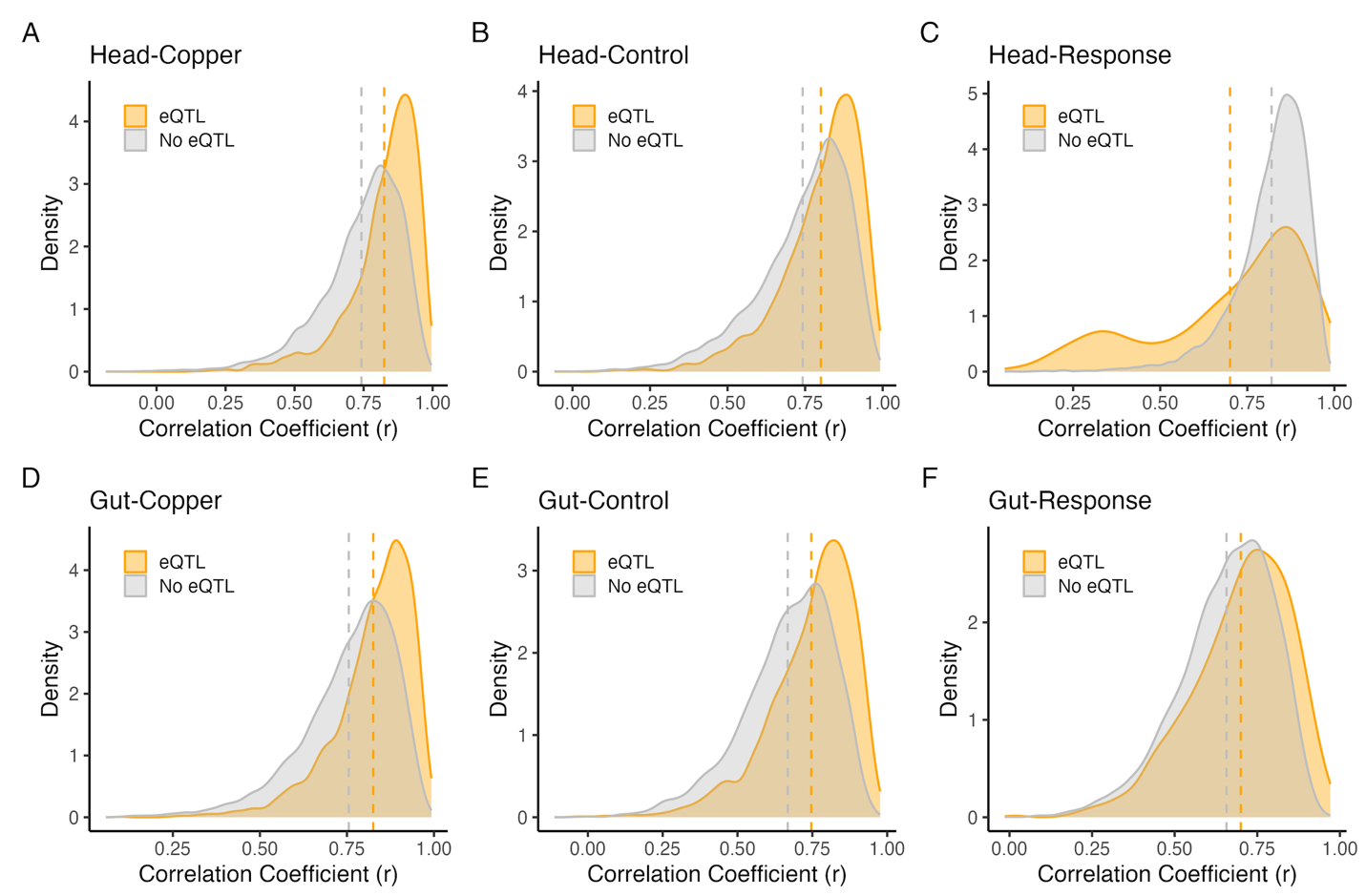


**Figure 5.** Genome-wide LOD scores were similar between the 1% and 2% corrected datasets for each tissue and treatment. Head-Copper, -Control, and -Response correlations are shown in A-C. Gut-Copper, -Control, and -Response correlations are shown in D-F. In each panel, orange highlights genome-wide LOD score correlations for genes that are associated with at least one eQTL in the 2% corrected data; grey highlights genes that were not associated with eQTL. Vertical color-coded lines indicate mean correlations for each group of genes.
