# Supplementary figures and images for: Gene expression variation underlying tissue-specific responses to copper stress in Drosophila melanogaster
Source: G3 (Bethesda). 2024 Jan 23;14(3):jkae015. doi: 10.1093/g3journal/jkae015 (PMC11021028; doi:10.1093/g3journal/jkae015)

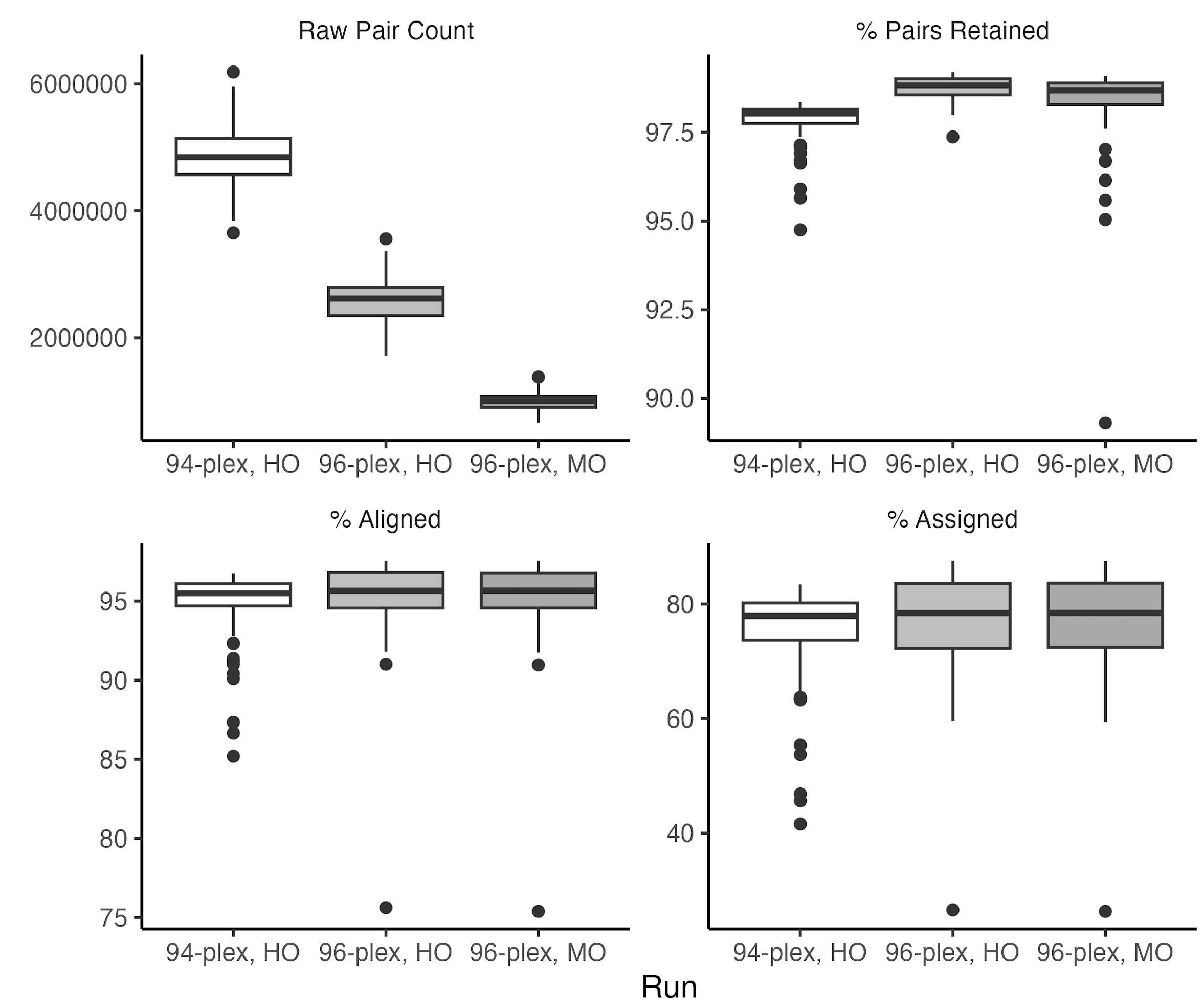

Supplement: jkae015_Supplementary_Data [file jkae015_supplementary_data.zip › Figure_S3_G3-2023-404710.jpg]

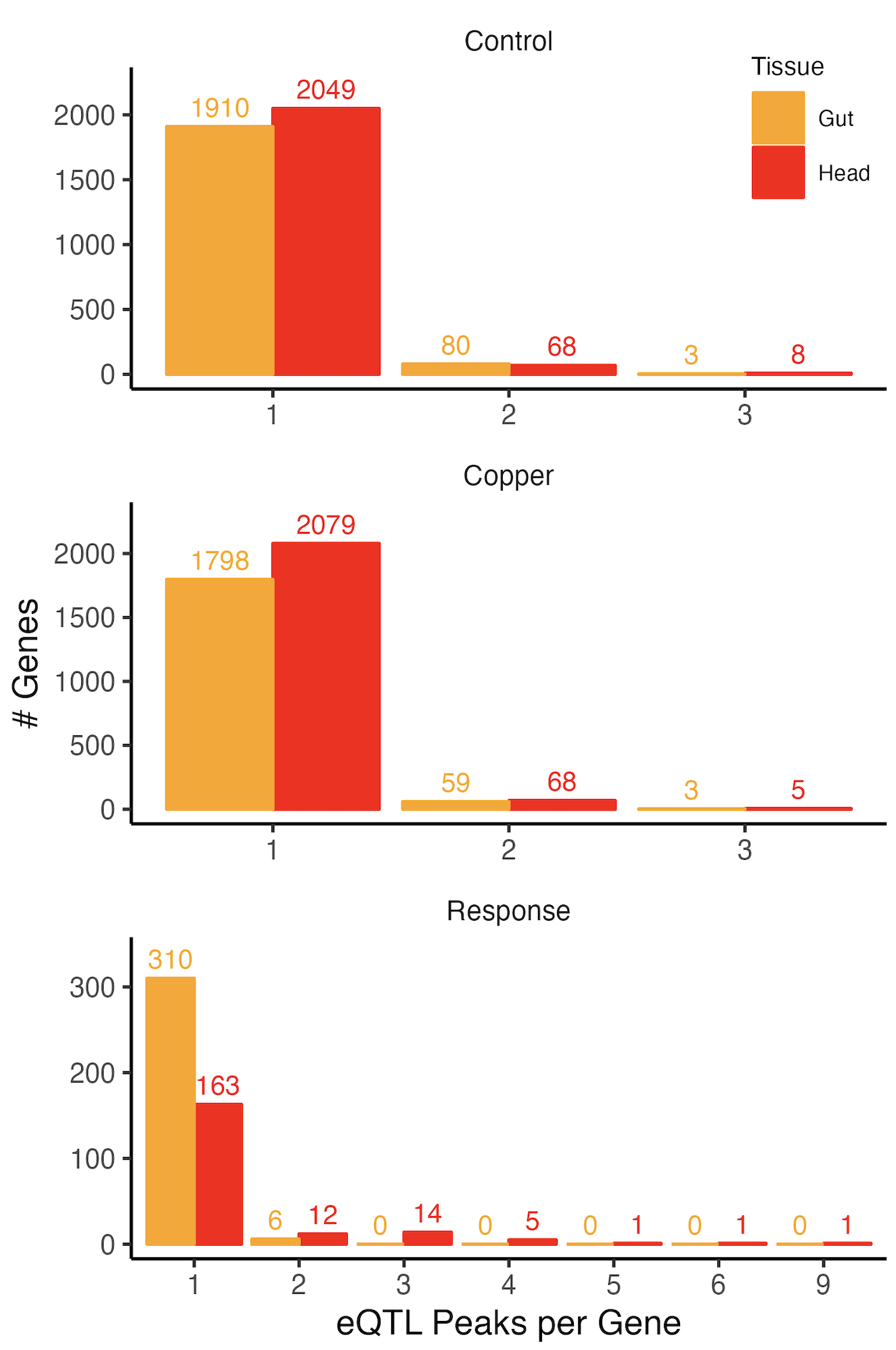

Supplement: jkae015_Supplementary_Data [file jkae015_supplementary_data.zip › Figure_S4_G3-2023-404710.jpg]

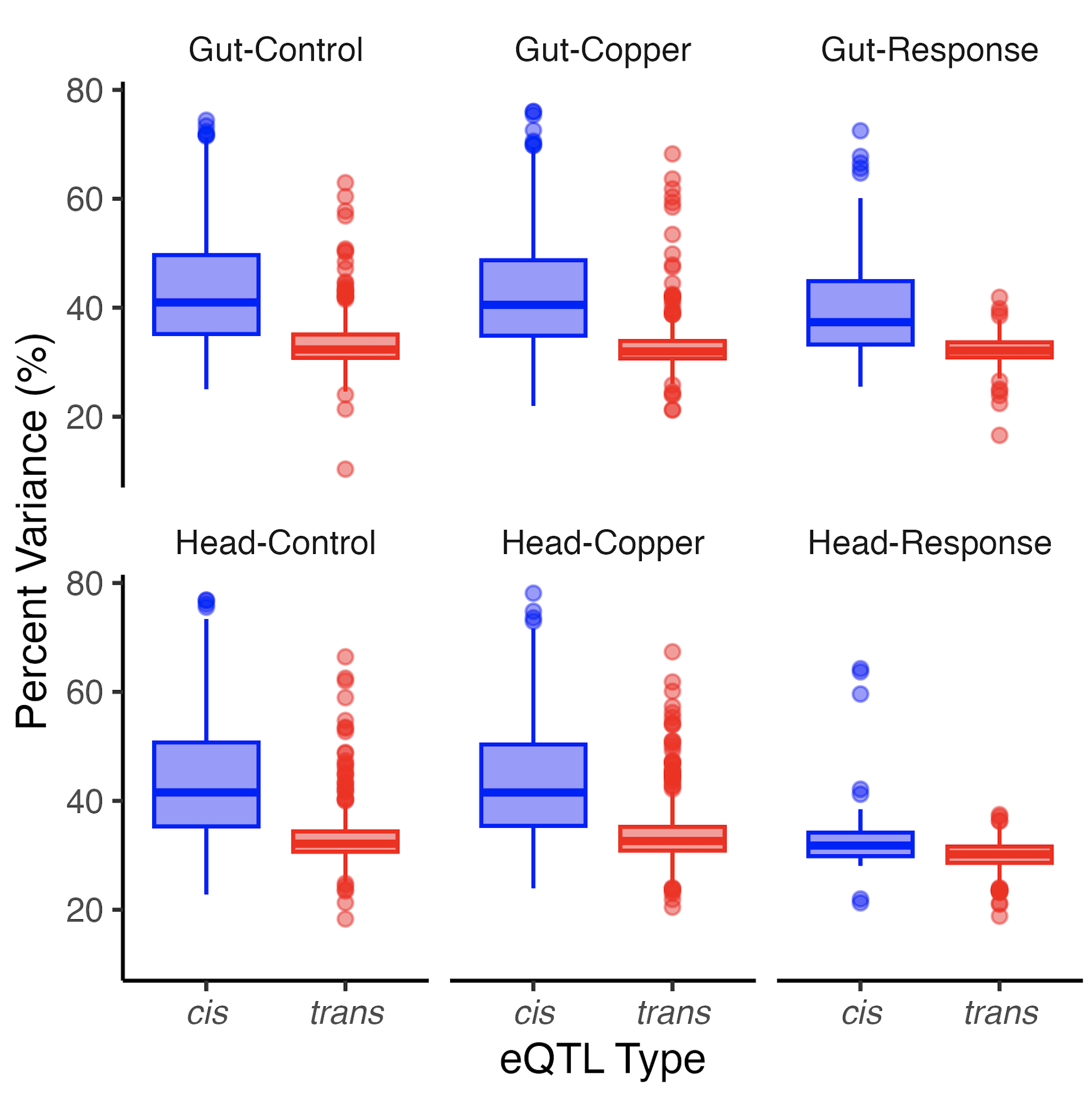

Supplement: jkae015_Supplementary_Data [file jkae015_supplementary_data.zip › Figure_S5_G3-2023-404710.jpg]

A

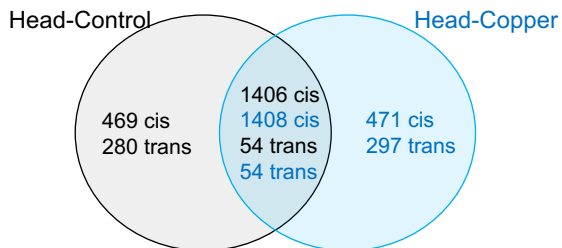

F

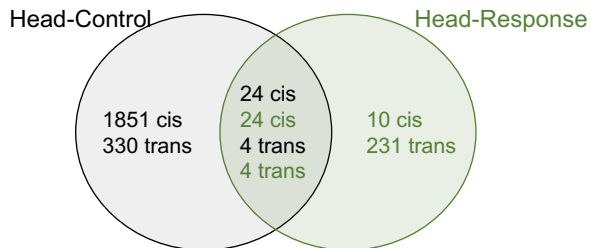

B

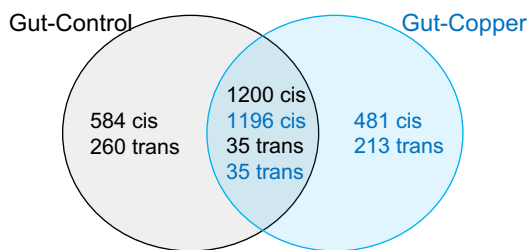

G

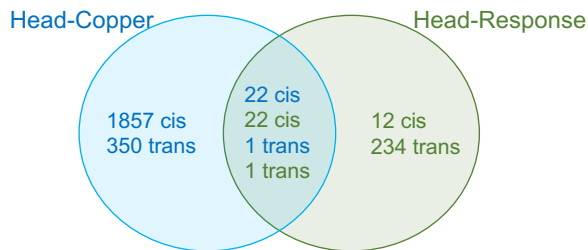

C

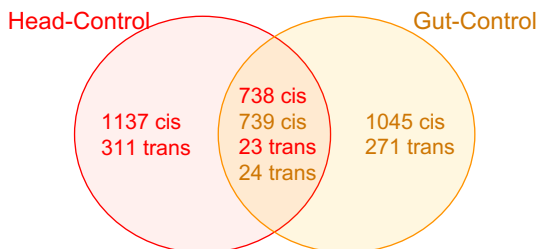

H

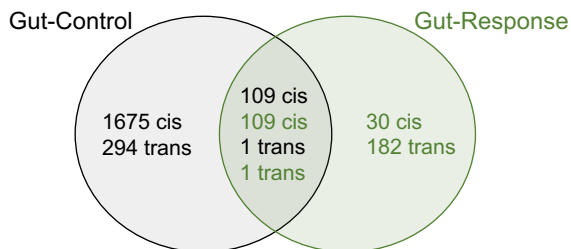

D

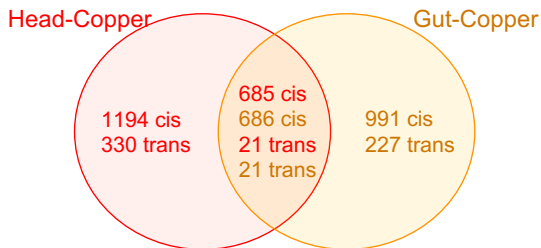

I

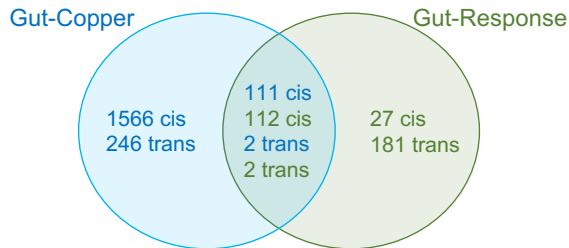

E

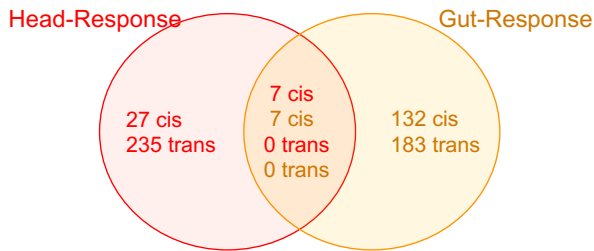

Supplement: jkae015_Supplementary_Data [file jkae015_supplementary_data.zip › Figure_S6_G3-2023-404710.pdf]

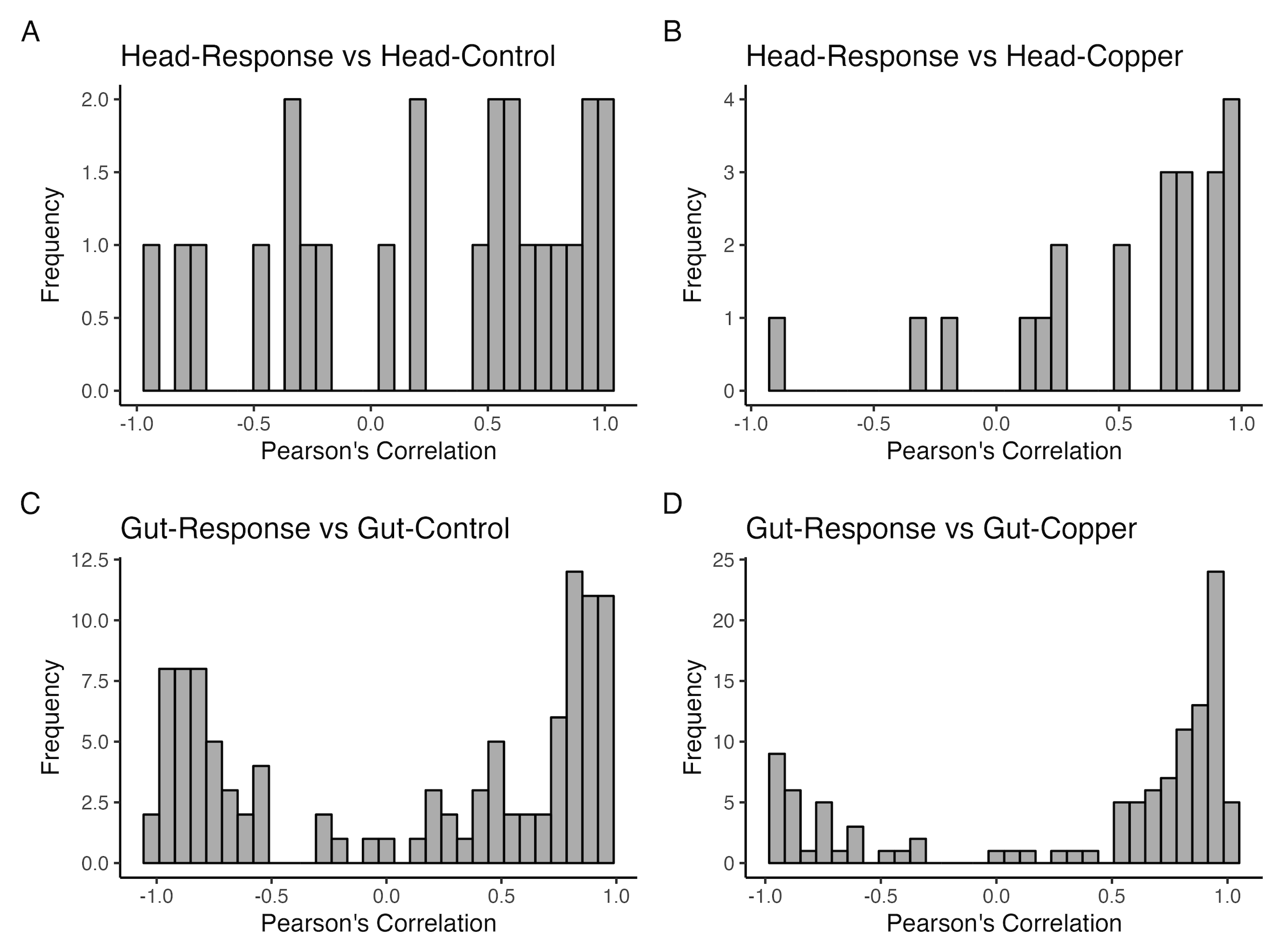

Supplement: jkae015_Supplementary_Data [file jkae015_supplementary_data.zip › Figure_S7_G3-2023-404710.jpg]

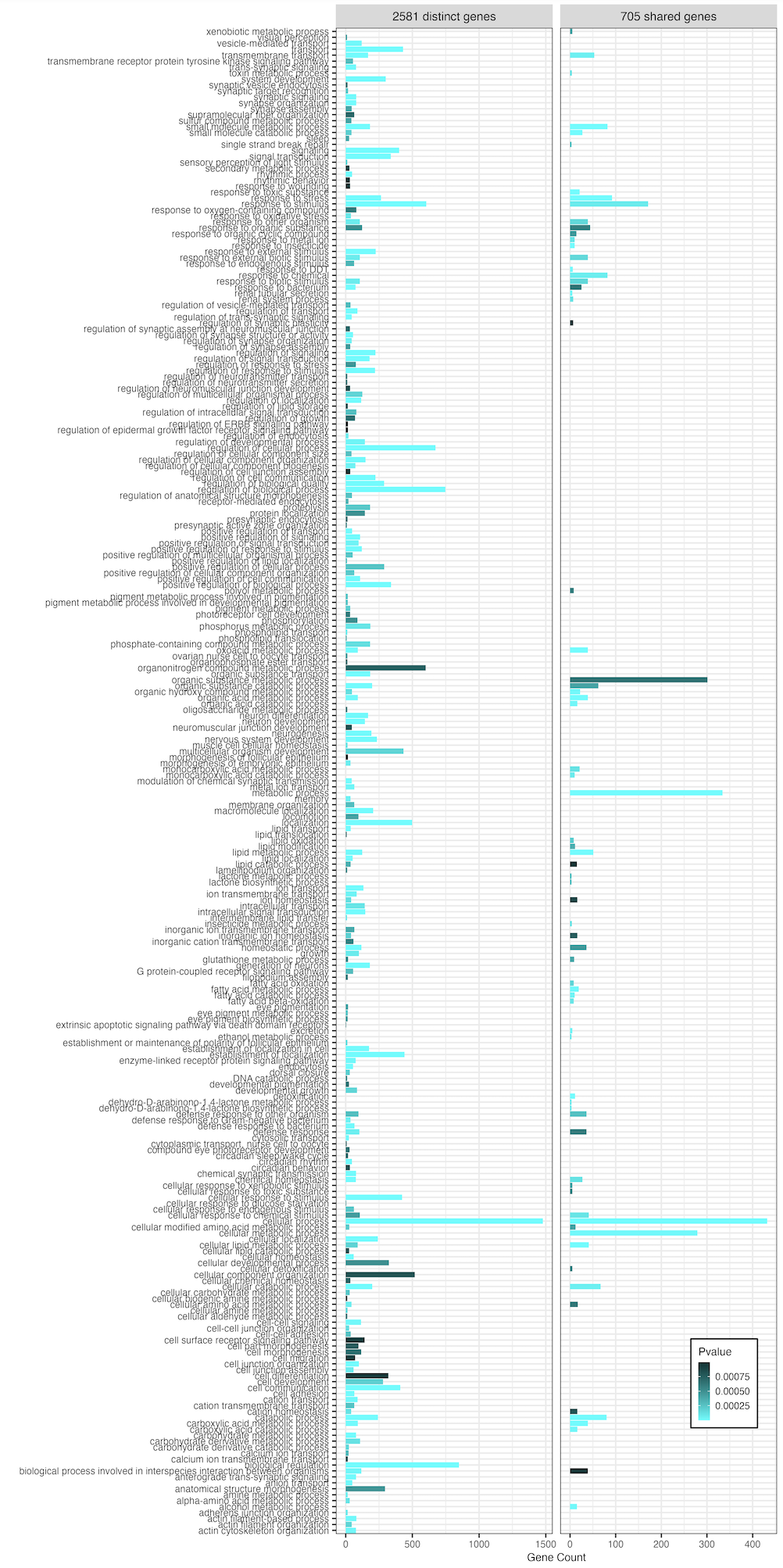

Supplement: jkae015_Supplementary_Data [file jkae015_supplementary_data.zip › Figure_S8_G3-2023-404710.jpg]

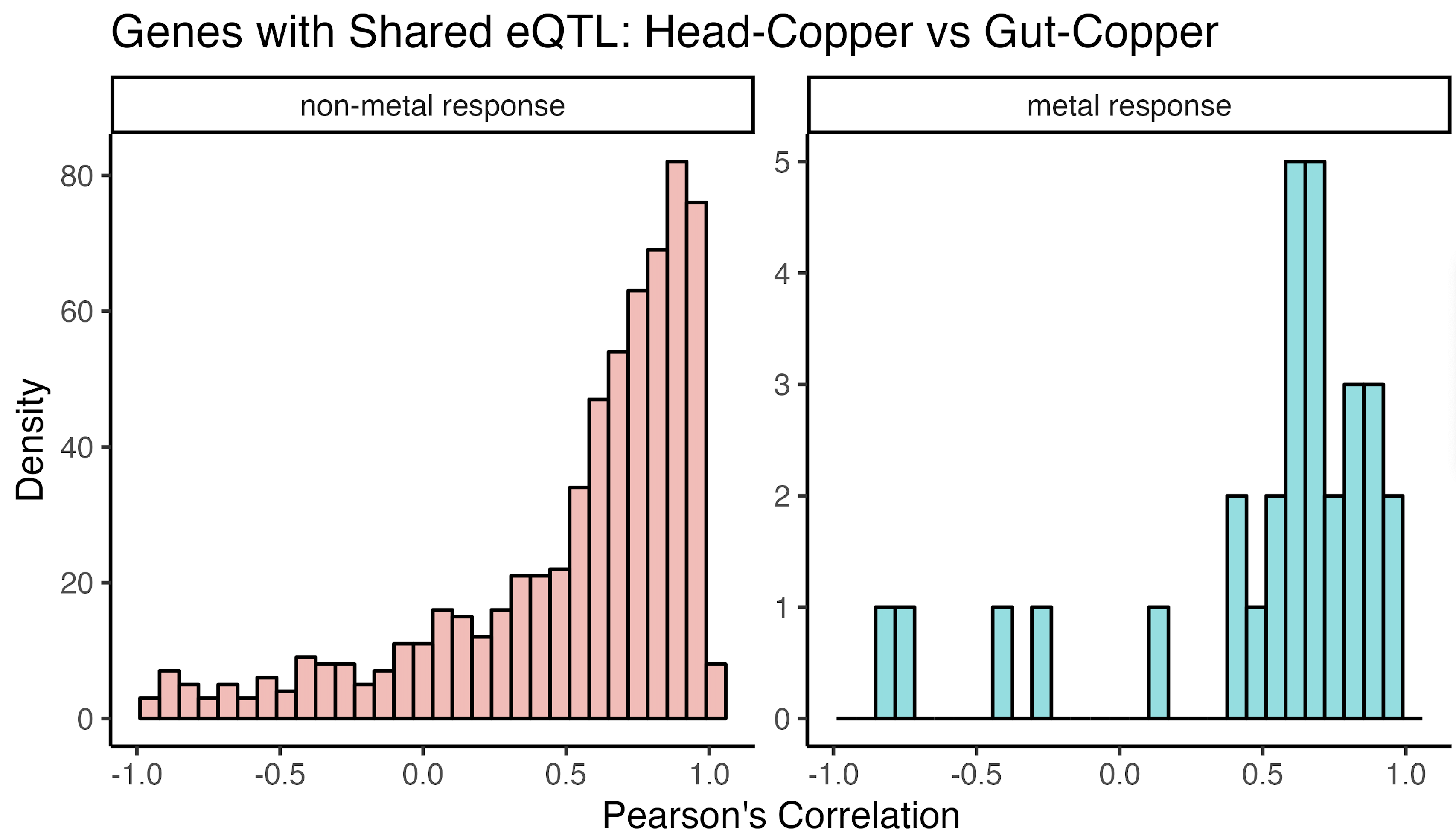

Supplement: jkae015_Supplementary_Data [file jkae015_supplementary_data.zip › Figure_S9_G3-2023-404710.jpg]

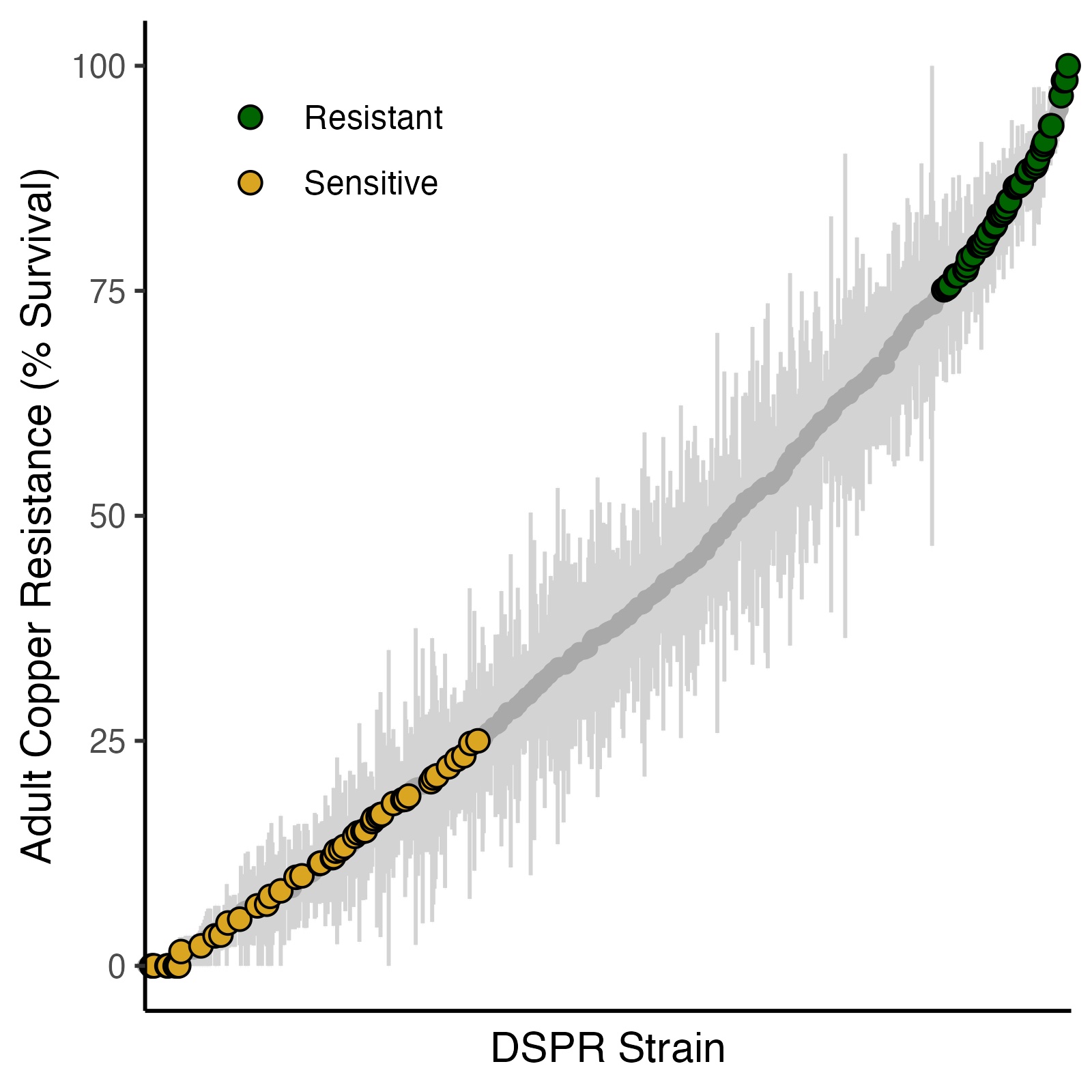

Supplement: jkae015_Supplementary_Data [file jkae015_supplementary_data.zip › Figure_S1_G3-2023-404710.jpg]

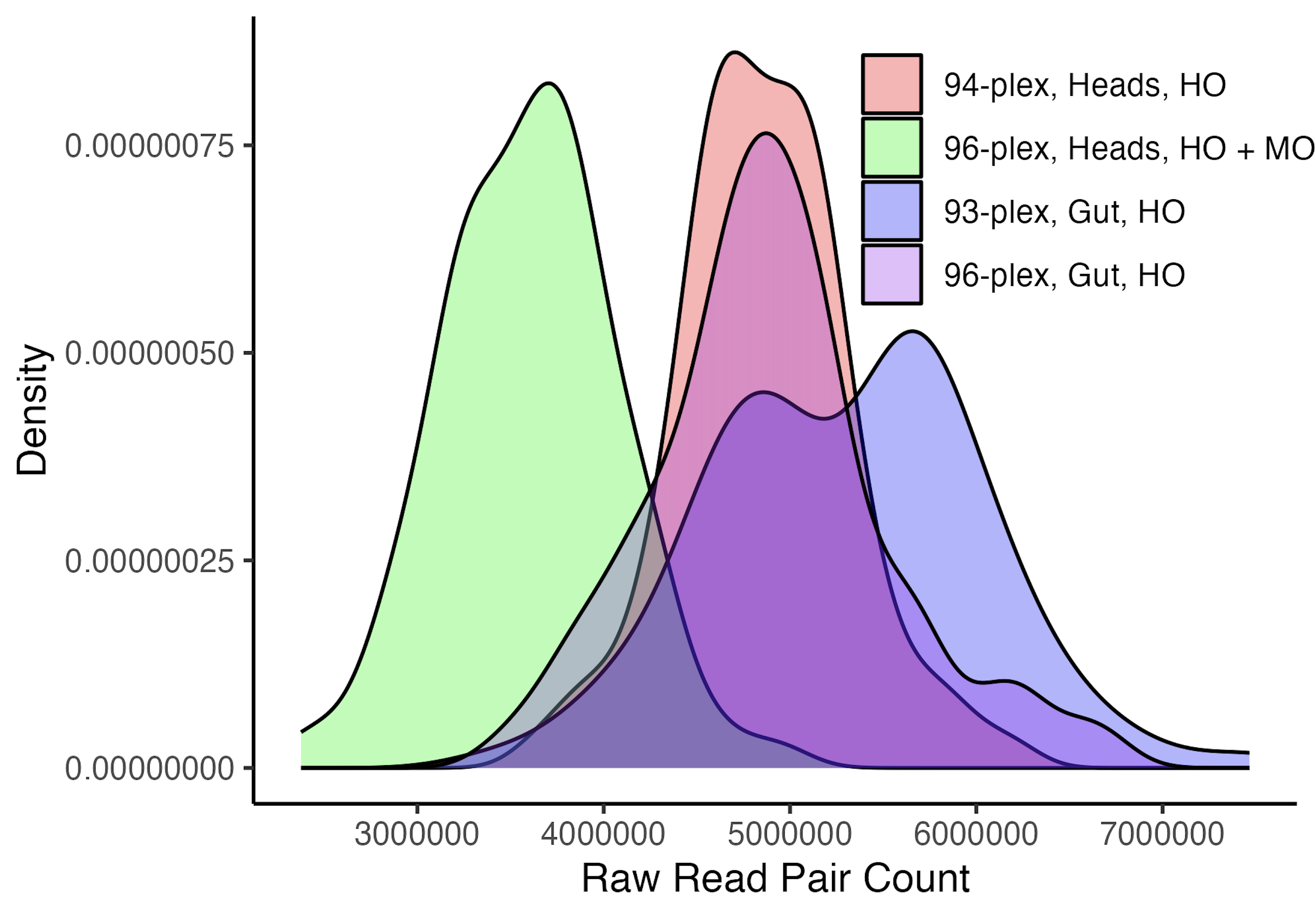

Supplement: jkae015_Supplementary_Data [file jkae015_supplementary_data.zip › Figure_S2_G3-2023-404710.jpg]
